# Primordial cardiomyocytes orchestrate myocardial morphogenesis and vascularization but are dispensable for regeneration

**DOI:** 10.7554/eLife.110256

**Published:** 2026-09-08

**Authors:** Jisheng Sun, Lu Chen, Jinhu Wang

**Affiliations:** 1 Cardiology Division, Department of Medicine, Emory University Atlanta United States; Boston College United States; Max Planck Institute for Heart and Lung Research Germany

**Keywords:** zebrafish, heart delopment, regeneration, cardiomyocyte, primordial cardiomyocyte, phlda2, Zebrafish

## Abstract

The vertebrate heart is composed of heterogeneous cardiomyocyte (CM) populations; however, the roles of distinct CM subpopulations in heart development and repair remain poorly defined. Here, using single-cell RNA sequencing analysis of adult zebrafish heart, we identified a unique CM subpopulation marked by the expression of *phlda2*, which is associated with anaerobic metabolism and different from mature CMs, which are enriched for oxidative phosphorylation genes. We demonstrated that *phlda2*^+^ cells constituted a primordial CM compartment localized between compact and trabecular muscles. Genetic ablation of *phlda2*^+^ CMs during development severely disrupted heart morphogenesis, leading to defective myocardial trabeculation and compaction, and impaired coronary vascularization. Surprisingly, despite their essential roles in development, the depletion of *phlda2*^+^ CMs didn’t impair myocardial restoration and revascularization following ventricular resection. We found that this was probably due to the limited regenerative capacity of the primordial CMs themselves, as they failed to regenerate after either surgical amputation or genetic ablation. Our findings identify primordial CMs as an organizer for heart morphogenesis but not essential for regeneration, revealing a fundamental difference between developmental and regenerative programs in the vertebrate heart.

## Introduction

The vertebrate myocardium is not a homogeneous tissue but consists of cardiomyocyte (CM) subpopulations that are functionally and molecularly different (Litviňuková et al., 2020; Anto Michel et al., 2022). Recent single-cell transcriptomic studies have revealed this cellular heterogeneity in both mammalian and zebrafish hearts, showing clusters of CMs with specialized metabolic, structural, and contractile properties (Wang et al., 2021; Honkoop et al., 2019; Forman-Rubinsky et al., 2025; Hu et al., 2022; Li et al., 2025). Although these studies provide the evidence of cellular diversity of the myocardium, the functional significance of these CM subpopulations during heart development and disease remains a central question in cardiac biology.

Humans, like all mammals, lack the natural ability to efficiently replace lost myocardium with new contractile tissue, a deficiency that results in the leading cause of morbidity and mortality (Uygur and Lee, 2016; Porrello et al., 2011; Laflamme and Murry, 2011). However, zebrafish possess a remarkable capacity to effectively regenerate the lost CMs after injury (Poss et al., 2002; Wang et al., 2011; González-Rosa et al., 2011; Chablais et al., 2011; Kikuchi et al., 2010; González-Rosa et al., 2017), facilitated by non-myocardial cells, including epicardial cells (Wang et al., 2015; Sun et al., 2022b), immune cells (Bruton et al., 2022; Peterson et al., 2024), endothelial cells (Marín-Juez et al., 2016; Harrison et al., 2019; Gancz et al., 2020), and nerve cells (Mahmoud et al., 2015), making zebrafish an excellent model for exploring natural heart regeneration. Previous studies have shown that spared CMs dedifferentiate and proliferate to replace the lost myocardium (Kikuchi et al., 2010; Jopling et al., 2010; Porrello et al., 2011). However, it remains unclear whether CM subpopulations contribute equally to myocardial restoration. Furthermore, although the regeneration process has been generally accepted to recapitulate the developmental program (Ross Stewart et al., 2022), it remains unknown whether all CM subpopulations play similar roles in heart morphogenesis and regeneration. Therefore, identifying and characterizing the roles of CM subpopulations are critical for understanding the mechanisms of heart development and repair.

Adult zebrafish myocardium is composed of three main types of CMs with different spatial arrangements: the inner mass of trabecular CMs, the outer layer of compact CMs, and a single-cell-thick layer of primordial CMs that are located beneath the compact layer (Gupta and Poss, 2012; Lafontant et al., 2013; Hu et al., 2001). While the trabecular and compact muscles have been extensively studied (Staudt and Stainier, 2012; Han et al., 2016; Gupta and Poss, 2012; Gupta et al., 2013; Liu et al., 2010), the function of primordial CMs remains elusive due to the lack of genetic tools for their manipulation. Primordial CMs are thought to retain immature characteristics (Tsedeke et al., 2021), but their molecular identity and functional contributions to myocardial morphogenesis and regeneration have not been fully elucidated. Here, we performed single-cell RNA sequencing (scRNA-seq) analysis of adult zebrafish heart to define CM subpopulations and investigated the contribution of primordial CMs to myocardial morphogenesis and regeneration. Our studies reveal that primordial CMs are an essential organizer for myocardial morphogenesis and coronary vascularization, but not required for myocardial restoration and revascularization, highlighting that regeneration goes beyond the reactivation of developmental programs.

## Results

### Characterization of CM heterogeneity in adult zebrafish heart

**Figure 1.**
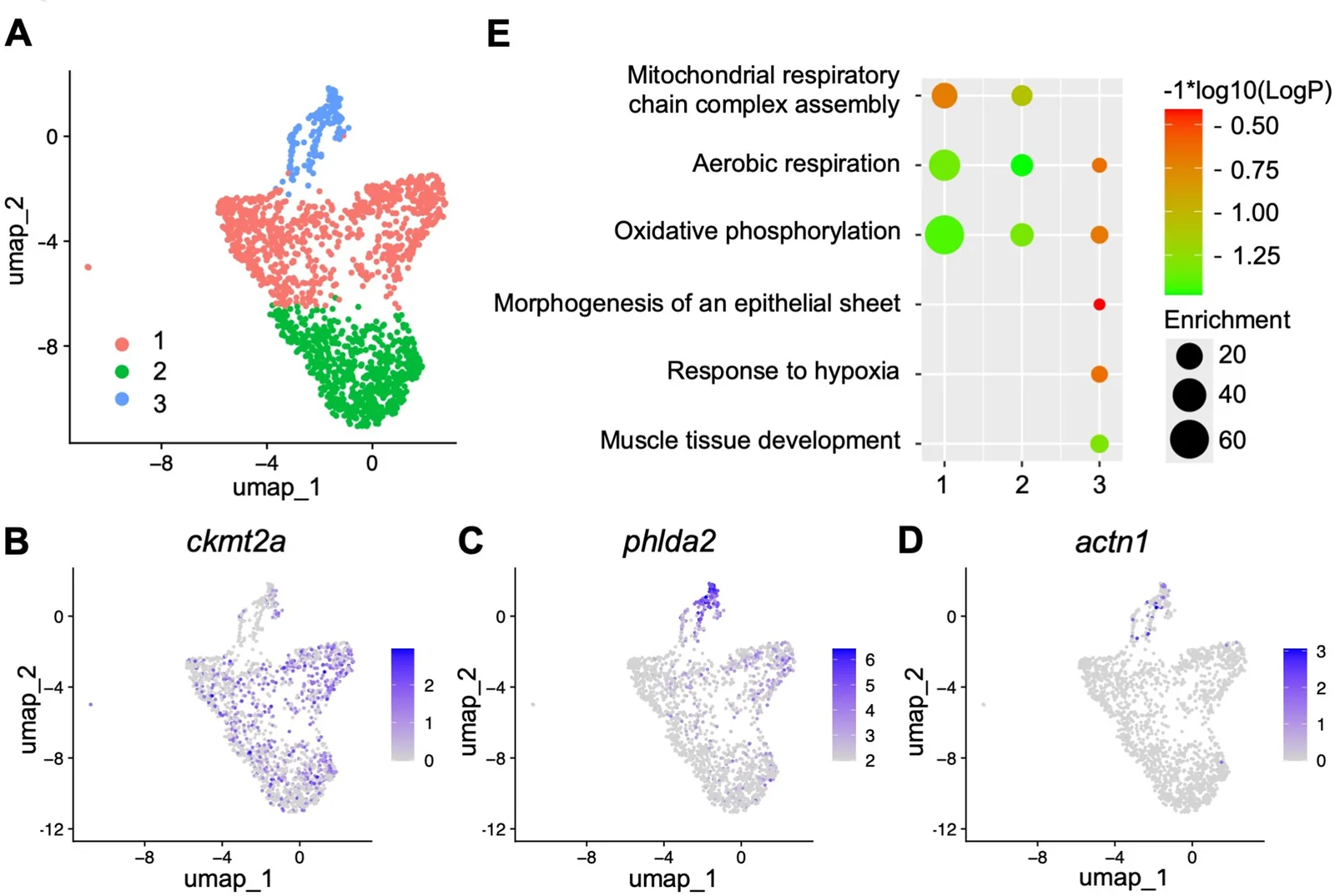
Single-cell RNA sequencing (scRNA-seq) experiments reveal distinct cardiomyocyte clusters in adult zebrafish hearts. (**A**) Uniform manifold approximation and projection (UMAP) clustering of *cmlc2*^+^ single cells from adult hearts. (**B–D**) Feature plot of *ckmt2a* (**B**), *phlda2* (**C**), *actn1* (**D**) expression in cardiomyocyte clusters of adult hearts. (**E**) Identification of cardiomyocyte clusters based on Gene Ontology analysis.

To characterize the cellular heterogeneity of adult zebrafish myocardium, we performed scRNA-seq with isolated CMs. The cells activating the regulatory sequences of the CM marker *cmlc2* were purified from the hearts of adult cmlc2*:EGFP* animals (Kikuchi et al., 2010). We applied stringent quality filtering and discarded a small number of *EGFP*^−^/*cmlc2*^−^ cells and obtained high-quality transcriptomes of 1668 CMs. The expression of cmlc2 transcripts and EGFP reporter signal in the single-cell dataset further confirmed the enrichment of CMs (Figure 1—figure supplement 1A and B). Unsupervised clustering of these cells identified three different clusters (Figure 1A), each with a distinct gene expression pattern. Clusters 1 and 2 showed an enrichment of genes associated with mature CMs, such as *ckmt2a* (Figure 1B) and *ckmt2b* (Figure 1—figure supplement 1C Tsedeke et al., 2021). However, due to their significant overlap, it remains unclear whether these two clusters represent distinct cellular identities of CMs. In contrast, Cluster 3 was marked by prominent expression of *phlda2* (Figure 1C), as well as *actn1* (Figure 1D), indicative of an immature cellular state (Tsedeke et al., 2021), and *notch3* (Figure 1—figure supplement 1D)*,* reflecting elevated Notch signaling activity (Han et al., 2016). To further validate their spatial distribution, analysis of previously published spatial transcriptomic data (Li et al., 2025) revealed that *phlda2*, *actn1*, and *notch3* were predominantly expressed in the outer region of the heart (Figure 1—figure supplement 2). To gain insights into the biological characteristics of these clusters, we performed Gene Ontology (GO) enrichment analysis with specifically enriched transcripts in each CM cluster (Figure 1E). Clusters 1 and 2 were enriched for GO terms related to aerobic respiration and oxidative phosphorylation, suggesting the mature CMs efficiently produce the energy to sustain the constant contraction (Neubauer, 2007; Garbern and Lee, 2021). Notably, Cluster 2 showed additional enrichment for pathways involved in mitochondrial respiratory chain assembly, ATP synthesis, TCA cycle, and ribosome biogenesis, suggesting a relatively higher metabolic and biosynthetic activity state. In contrast, Cluster 1 was enriched for GO terms associated with mitochondrial stress responses, protein degradation, and cytoprotective pathways, indicating a stress-adapted CM state. Cluster 3 showed reduced enrichment of metabolic pathways, consistent with an immature metabolic profile, and was further enriched for genes involved in muscle development and epithelial morphogenesis, suggesting a role in cardiac morphogenesis and tissue organization.

**Figure 1—figure supplement 1.**
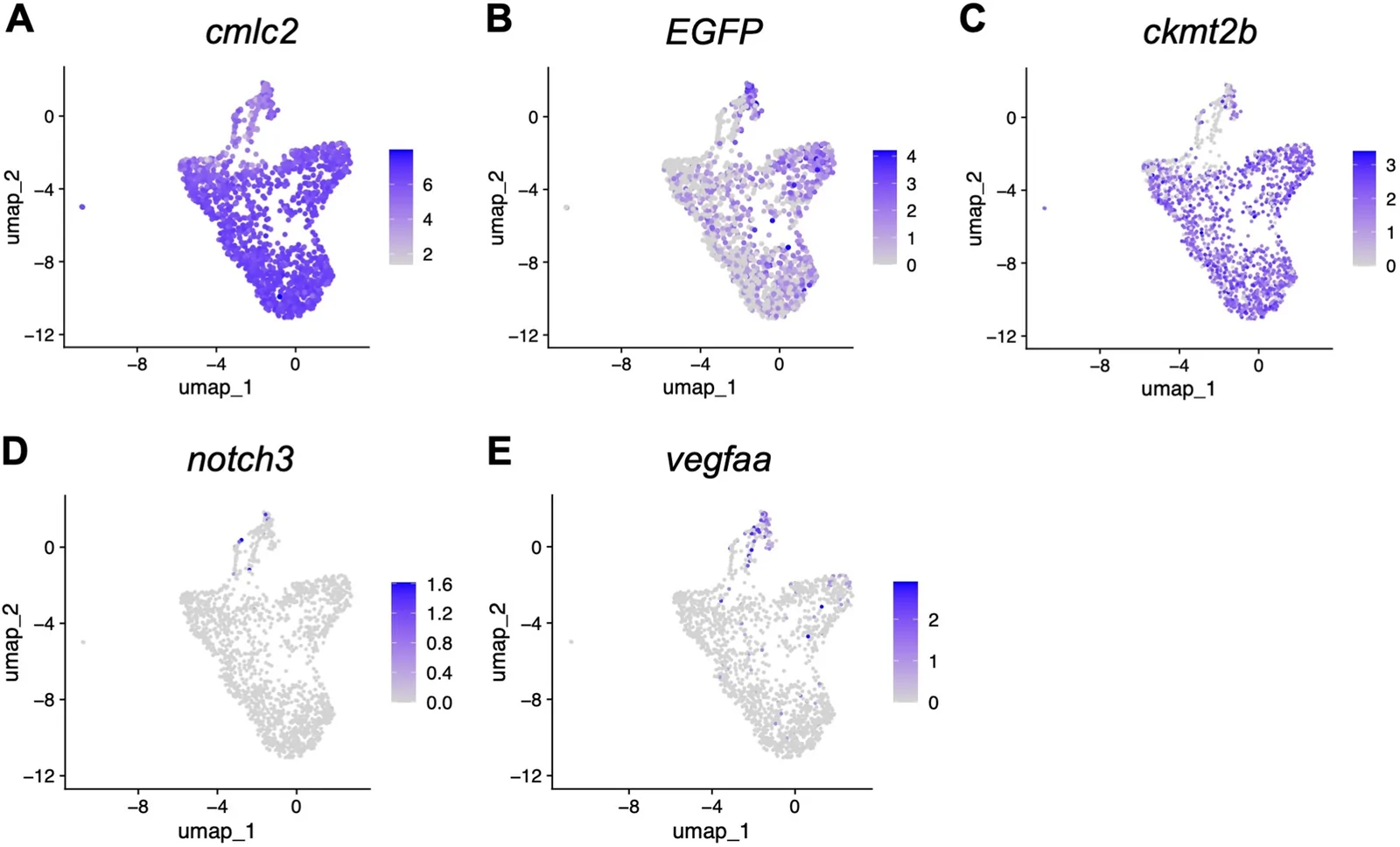
Feature plot of *cmlc2* (**A**), *EGFP* (**B**), *ckmt2b* (**C**), *notch3* (**D**), *vegfaa* (**E**) expression in cardiomyocyte clusters of adult hearts.

**Figure 1—figure supplement 2.**
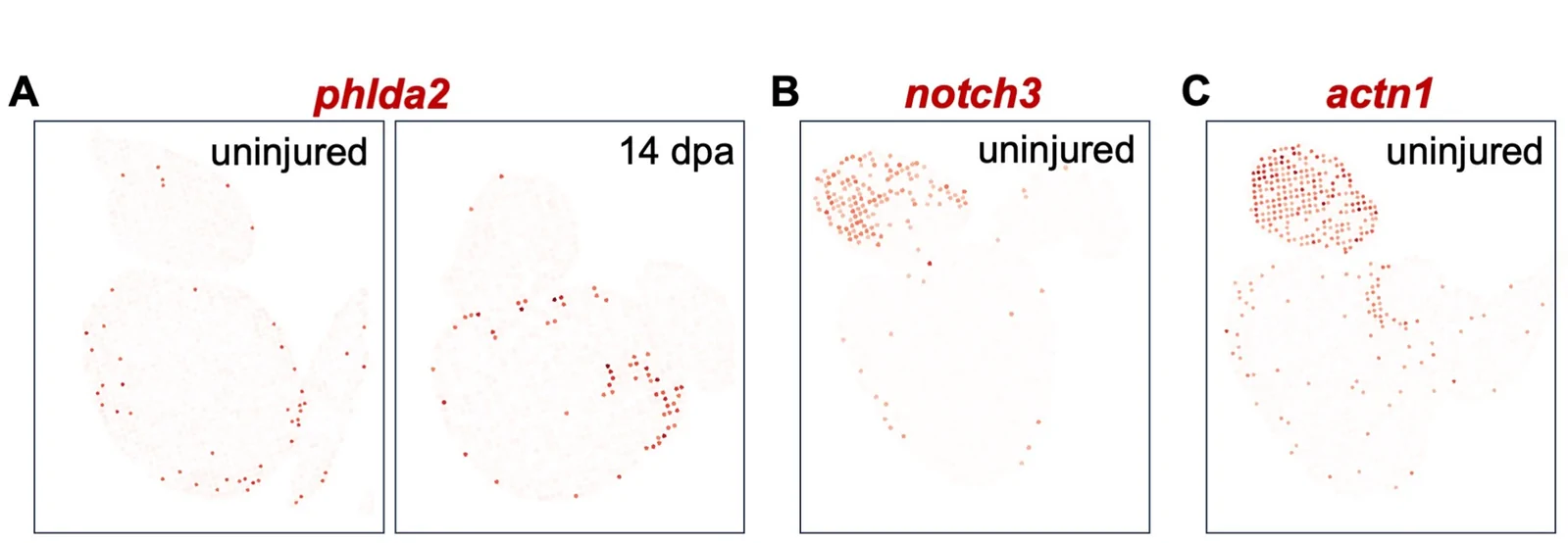
Spatial visualization of imputed genes using a published zebrafish heart regeneration spatial transcriptomic dataset. (**A**) Spatial visualization of imputed expression of *phlda2* in uninjured (left) and regenerated hearts at 17 days post-amputation (dpa) (right). (**B–C**) Spatial visualization of imputed expression of *notch3* (**B**) and *actn1* (**C**) in uninjured hearts.

### The *phlda2*:EGFP^+^ cells represent primordial CMs in adult zebrafish hearts

**Figure 2.**
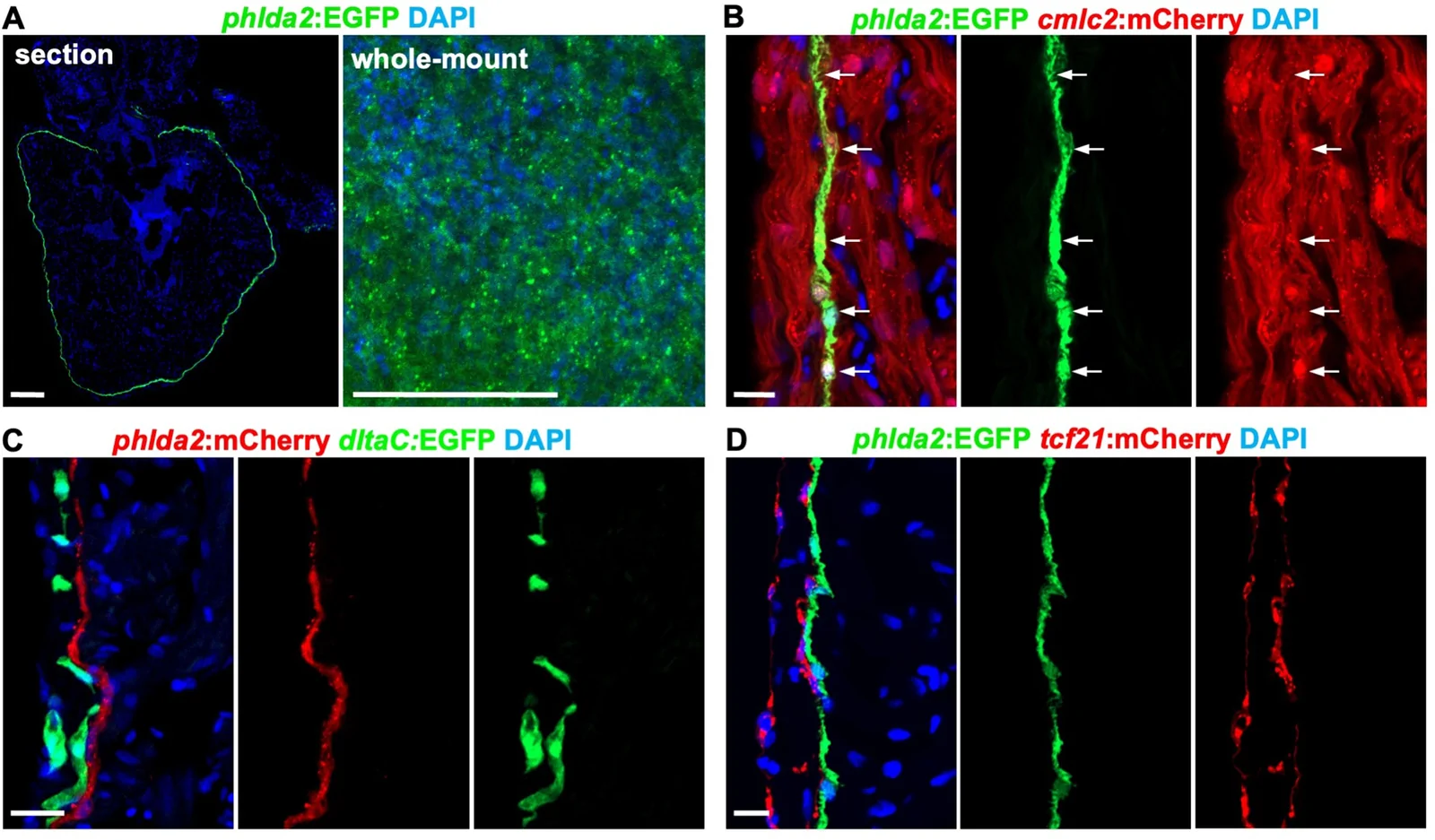
*phlda2*:EGFP^+^ cells specifically label primordial cardiomyocytes in adult zebrafish hearts. (**A**) *phlda2*^+^ cells visualized in whole cardiac sections and whole-mount heart from adult *phlda2:EGFP* animals. n=8. Scale bar, 100 µm. (**B**) Confocal slices indicating *phlda2*^+^ cells in uninjured *phlda2:EGFP;cmlc2:mCherry* ventricles. White arrows represent *phlda2*^+^/*cmlc2*^+^ cells. n=8. Scale bar, 10 µm. (**C**) Confocal slices indicating *phlda2*^+^ cells in adult *phlda2:mCherry;deltaC:EGFP* ventricles. n=12. Scale bars, 20 µm. (**D**) Confocal slices indicating *phlda2*^+^ cells in adult *phlda2:EGFP;tcf21:mCherry* ventricles. n=10. Scale bars, 10 µm. All data are representative of two independent experiments.

Primordial layer is a single-CM thickness, characteristics of the embryonic ventricle, and high notch activity (Gupta and Poss, 2012; Han et al., 2016; Tsedeke et al., 2021). To determine whether Cluster 3 is primordial CMs, we generated *phlda2:EGFP* and *phlda2:mCherry-NTR* BAC transgenic animals, containing sequences of 64,684 bp upstream of the *phlda2* translation initiation codon and 51,710 bp downstream of the stop codon. We observed prominent fluorescence signals in the ventricular wall covering the surface of the ventricle (Figure 2A). As predicted by scRNA-seq result, *phlda2*:EGFP^+^ expression occupied a subset of cells marked by *cmlc2:*mCherry^+^ fluorescence in adult hearts (Figure 2B), indicating that *phlda2*^+^ cells are CMs. Moreover, *phlda2*-directed fluorescence signals did not colocalize with reporter transgenes marking coronary vessels or epicardial cells (Wang et al., 2015; Sun et al., 2022a; Figure 2C and D). Examination of juvenile hearts revealed similar expression in the primordial CM layer (Figure 2—figure supplement 1A), indicating that *phlda2* transgenic reporters label the primordial cardiac wall throughout the development.

**Figure 2—figure supplement 1.**
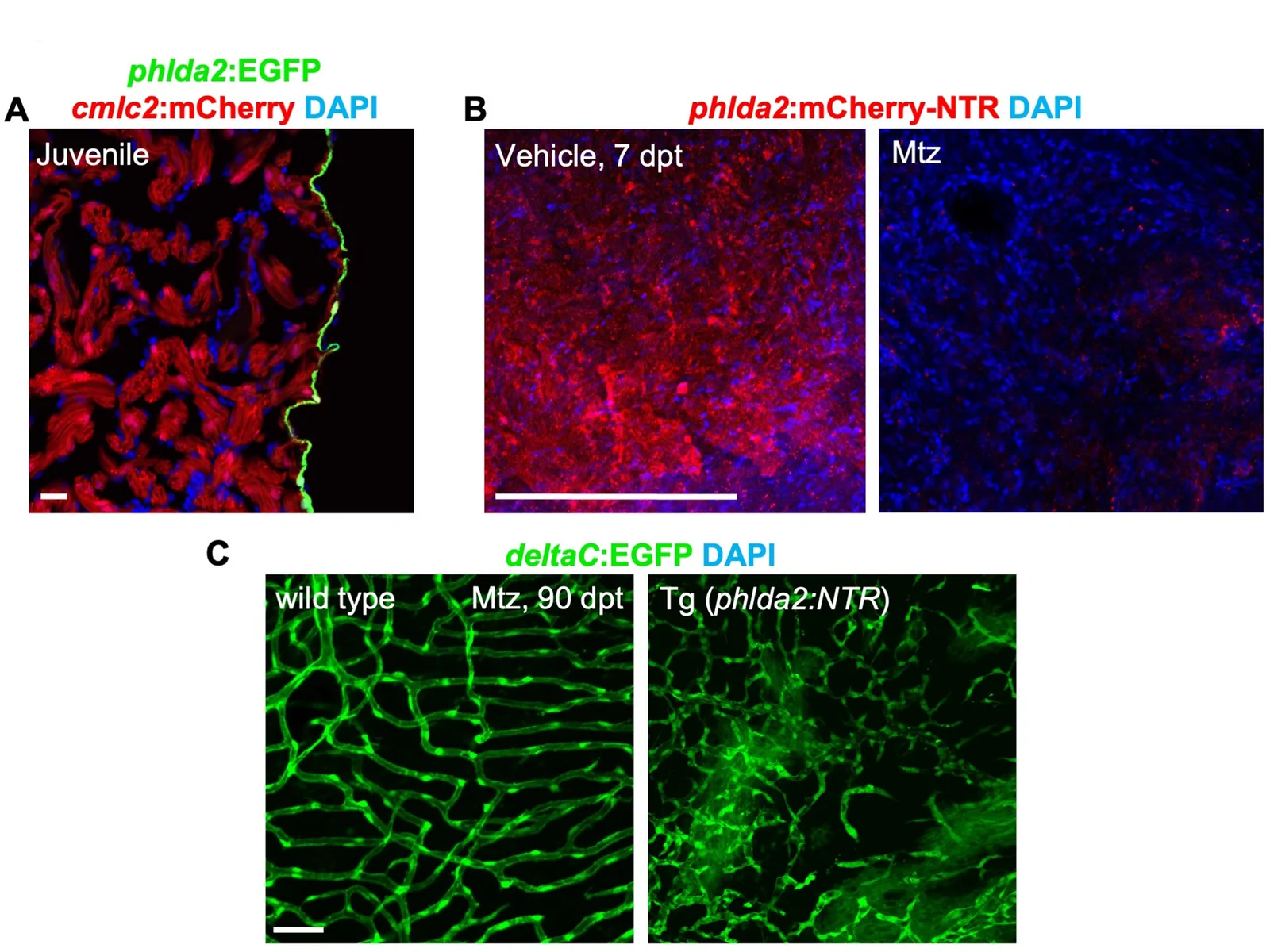
Validation of *phlda2*^+^ cardiomyocyte ablation in juvenile hearts. (**A**) Confocal slices indicating *phlda2*^+^ cells in juvenile *phlda2:EGFP; cmlc2:mCherry* ventricles. n=10. Scale bar, 20 µm. (**B**) Visualization of *phlda2*^+^ cells in whole-mounted adult *phlda2:mCherry-NTR* hearts after vehicle or Mtz treatment. n=12. Scale bars, 20 µm. (**C**) Coronary vessels in whole-mounted *deltaC:EGFP* and *deltaC:EGFP;phlda2:mCherry-NTR* hearts. Animals were treated with Mtz during the juvenile stage and analyzed at 90 days post-treatment (dpt). n=5. Scale bars, 50 µm. All data are representative of two independent experiments.

### Primordial CMs are required for morphogenesis of myocardium and coronary vascularization

**Figure 3.**
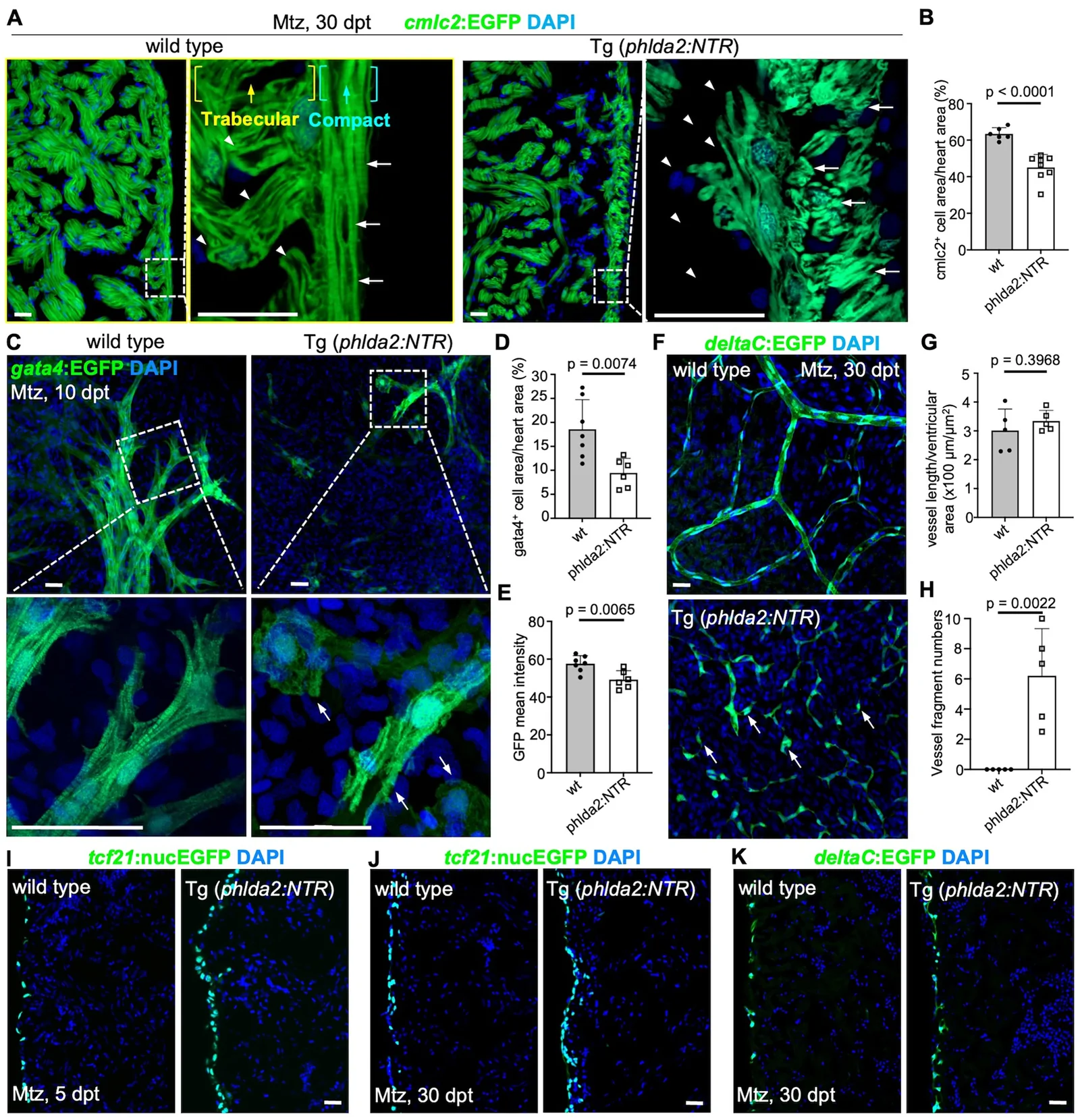
Primordial cardiomyocytes are required for morphogenesis of myocardium and coronary vascularization. (**A**) Compact and trabecular muscle in ventricular sections from Mtz-treated *cmlc2:EGFP* and *cmlc2:EGFP;phlda2:mCherry-NTR* animals. Animals were treated with Mtz during the juvenile stage and analyzed 30 days post-treatment (dpt). Boxed area is enlarged with high magnification. Arrows represent the compact muscle and arrowheads represent trabecular muscle. N=6–8. Scale bar, 20 µm. (**B**) Quantification of *cmlc2*^+^ cell area relative to heart area within 100 µm of the heart wall from experiments in (**A**). Mann-Whitney rank-sum test. (**C**) Visualization of *gata4*^+^ cardiomyocytes in whole-mounted juvenile *gata4:EGFP* and *gata4:EGFP;phlda2:mCherry-NTR* hearts after Mtz treatment. Boxed area is enlarged with high magnification. Arrowheads represent EGFP signals in *phlda2* cell-depleted hearts. n=6–7. Scale bars, 20 µm. (**D–E**) Quantification of the percentage of EGFP^+^ pixels (**D**) and EGFP intensity (**E**) on the ventricular surface from experiments in (**D**) and (**E**). Mann-Whitney rank-sum test. (**F**) Coronary vessels in whole-mounted *deltaC:EGFP* and *deltaC:EGFP;phlda2:mCherry-NTR* hearts. Animals were treated with Mtz during the juvenile stage and analyzed at 30 dpt. Arrows represent the broken vessel fragments. n=5. Scale bars, 20 µm. (**G–H**) Quantification of vessel length/heart area (**G**) and vessel fragments (**H**) on the ventricular surface from experiments in (**F**). Vessel length was measured within a fixed region of interest (ROI) after skeletonization of *deltaC:EGFP*^+^ vessels in ImageJ. (**I–J**) Visualization of *tcf21*^+^ epicardial cells in sectioned *tcf21:nucEGFP* and *tcf21:nucEGFP;phlda2:mCherry-NTR* hearts. Animals were treated with Mtz during the juvenile stage and analyzed at 5 and 30 dpt. n=5. Scale bars, 20 µm. (**K**) Coronary vessels in sectioned *deltaC:EGFP* and *deltaC:EGFP;phlda2:mCherry-NTR* hearts. Animals were treated with Mtz during the juvenile stage and analyzed at 30 dpt. n=5. Scale bars, 20 µm. All data are representative of three independent experiments.

The zebrafish myocardium consists of an inner trabecular layer, a thin primordial layer, and an outer compact layer. Previous multiclonal studies revealed that primordial CMs can give rise to trabecular CMs, which later breach the primordial layer to contribute to the compact myocardium (Gupta and Poss, 2012). It was reported that nearly 60% of CMs within both the trabecular and outer myocardium originated from primordial cells labeled at the embryonic stage and analyzed at 21 days post-fertilization (dpf) (Pfefferli and Jaźwińska, 2017). However, these observations only demonstrate a potential lineage relationship. Whether this cell population is required for the formation or structural integrity of these myocardial layers remains unknown. A major barrier to addressing this question has been the lack of genetic tools for the specific ablation of primordial cells. To address this, we used the bacterial NTR system for inducible cell ablation (Curado et al., 2008). We generated a new transgenic line and incubated *phlda2:mCherry-NTR* adults with the prodrug Mtz (Curado et al., 2008). This treatment depleted approximately 96.9% of ventricular *phlda2*^+^ cells without apparent effects on animal survival (Figure 2—figure supplement 1B). To address the role of *phlda2*^+^ cells during heart development, we performed the following experiments using a standardized Mtz treatment protocol (see Methods), with identical treatment conditions applied to 7–8 weeks post-fertilization (wpf) juvenile zebrafish. We incubated juvenile *phlda2:mCherry-NTR;cmlc2:EGFP* animals and control *cmlc2:EGFP* siblings for 3 days with Mtz, and then assessed the hearts for histological analysis at 10 and 30 days post-treatment (dpt). We found primordial CM depletion led to structural abnormality in the heart. The compact myocardium was disorganized compared with controls, and trabecular muscle formation was severely impaired, with an approximately 54.7% reduction in trabecular area, predominantly observed in regions adjacent to the ventricular wall (Figure 3A, B, Figure 3—figure supplement 1A). In juvenile zebrafish ventricles, *gata4*:EGFP^+^ CMs form the initial clones of compact muscle, which then gradually expand and converge with others to eventually encapsulate the ventricle and create a contiguous wall of compact muscle (Gupta et al., 2013). We analyzed hearts of *phlda2:mCherry-NTR;gata4:EGFP* fish and identified the ablation of primordial cells showed loss of sarcomere organization compared with control siblings at 10 dpt (Figure 3C, Figure 3—figure supplement 1B). Moreover, primordial cell-depleted hearts exhibited a 16.9% reduction in *gata4*:EGFP fluorescence intensity and a 48.9% reduction in the fluorescence area (Figure 3D and E).

**Figure 3—figure supplement 1.**
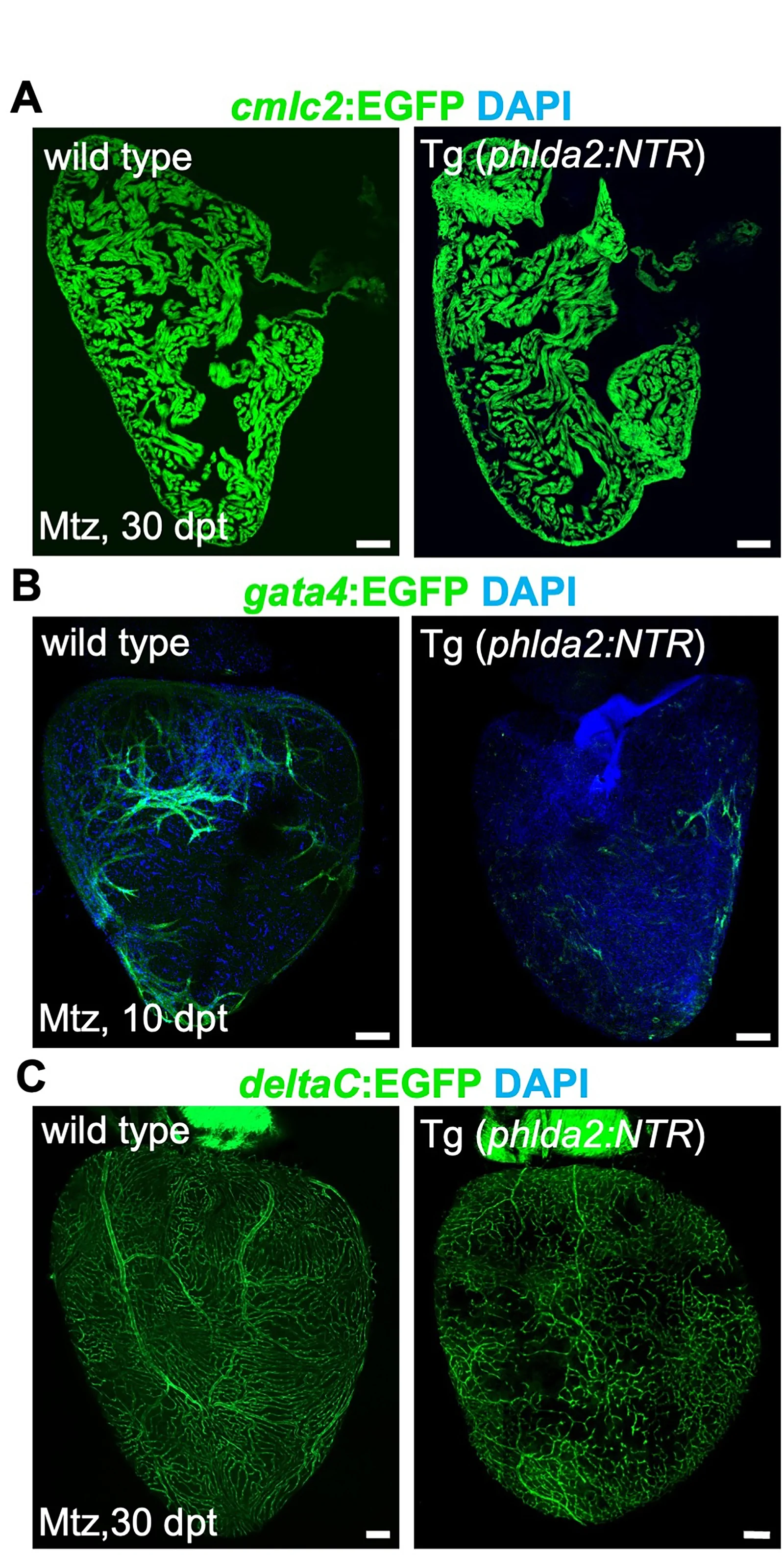
Low-magnification images showing the entire heart following juvenile-stage ablation of *phlda2*^+^ cardiomyocytes. (**A**) Compact and trabecular muscle in ventricular sections from Mtz-treated *cmlc2:EGFP* and *cmlc2:EGFP;phlda2:mCherry-NTR* animals. Animals were treated with Mtz during the juvenile stage and analyzed 30 days post-treatment (dpt). Scale bar, 100 µm. (**B**) Visualization of *gata4*^+^ cardiomyocytes in whole-mounted juvenile *gata4:EGFP* and *gata4:EGFP;phlda2:mCherry-NTR* hearts after Mtz treatment. Scale bars, 100 µm. (**C**) Coronary vessels in whole-mounted *deltaC:EGFP* and *deltaC:EGFP;phlda2:mCherry-NTR* hearts. Animals were treated with Mtz during the juvenile stage and analyzed at 30 dpt. Scale bars, 100 µm. All data are representative of two independent experiments.

During zebrafish heart development, the ventricle becomes vascularized after the compact myocardium has formed post-embryonically (Harrison et al., 2015). This process is closely associated with myocardial maturation and likely depends on proper organization of the compact layer to provide structural and molecular cues for vessel growth and integrity. To determine whether ablation of primordial CMs affects vascularization during heart development, we examined coronary vessel formation in *phlda2:NTR;deltaC:EGFP* fish, in which *deltaC:EGFP* has been utilized to visualize coronary endothelial cells (Sun et al., 2022a; Sun et al., 2023). We found that coronary vessels were fragmented and poorly developed in primordial cell-ablated hearts, although the total vessel length showed no reduction (Figure 3F–H, Figure 3—figure supplement 1C). Notably, these vascular abnormalities persisted at 90 dpt, indicating that the defects do not resolve during subsequent cardiac growth and maturation (Figure 2—figure supplement 1C). Because epicardial cells play critical roles in coronary vessel development, we next asked whether the vascular defects observed following primordial CM ablation were secondary to alterations in the epicardium. We found that *tcf21*^+^ epicardial cells revealed an increase rather than a decrease in epicardial cell abundance in primordial CM-ablated hearts (Figure 3I and J). These findings suggest that the impaired coronary vessel organization is unlikely to result from a reduction in epicardial cell number, although we cannot exclude the possibility that altered epicardial function or signaling contributes to the vascular phenotype. In addition, because primordial CMs form a continuous layer at the ventricular surface, we investigated whether their ablation permits abnormal invasion of epicardial cells or coronary vessels into the myocardium. However, we observed no evidence of ectopic localization of either cell population following primordial CM ablation (Figure 3J and K). Thus, loss of the primordial CM layer does not appear to disrupt tissue compartmentalization or permit abnormal cellular invasion into the myocardium. Together, these findings demonstrate that primordial CMs are required for proper myocardial growth and coronary vascularization during heart development.

### Primordial CMs are not essential for myocardial regeneration and coronary revascularization

**Figure 4.**
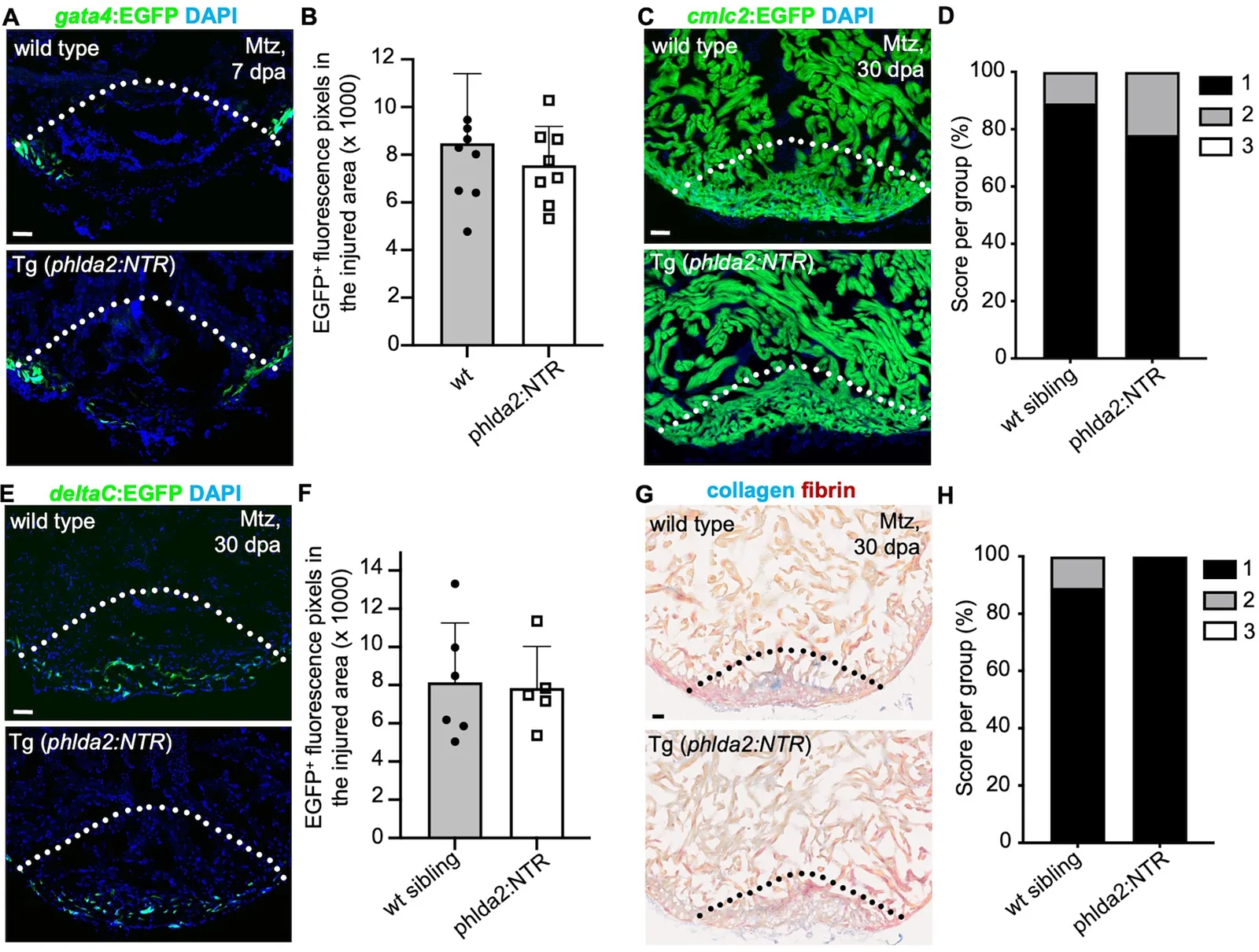
Primordial cardiomyocytes are not essential for myocardial regeneration and coronary revascularization. (**A**) Section images of Mtz-treated *gata4:EGFP* or *gata4:EGFP;phlda2:mCherry-NTR* ventricles at 7 days post-amputation (dpa) assessed for *gata4*:EGFP^+^ cardiomyocytes (CMs) in the injury site. Adult animals were treated with Mtz before amputation. Dashed line indicates amputation plane. N=8. Scale bars, 20 µm. (**B**) Quantification of EGFP^+^ pixels from experiments in (**A**). Scale bars, 20 µm. Mann-Whitney rank-sum test. (**C**) Section images of Mtz-treated *cmlc2:EGFP* or *cmlc2:EGFP;phlda2:mCherry-NTR* ventricles at 30 dpa assessed for muscle recovery. Adult animals were treated with Mtz before amputation. Dashed line indicates amputation plane. N=9–10. Scale bar, 20 µm. (**D**) Quantification of regeneration indices from experiments in (**C**). Myocardial regeneration is categorized as follows: 1=complete regeneration of a new myocardial wall; 2=partial regeneration; and 3=a strong block in regeneration. (**E**) Section images of Mtz-treated *deltaC:EGFP* or *deltaC:EGFP;phlda2:mCherry-NTR* ventricles at 30 dpa assessed for coronary vessels in the injury site. Adult animals were treated with Mtz before amputation. Dashed line indicates amputation plane. N=5–6. Scale bars, 20 µm. (**F**) Quantification of EGFP^+^ pixels from experiments in (**E**). Scale bars, 20 µm. Mann-Whitney rank-sum test. (**G**) Section images of Mtz-treated *wt siblings* or *phlda2:mCherry-NTR* ventricles at 30 dpa assessed for AFOG staining collagen/fibrin deposition in the injury site. Adult animals were treated with Mtz before amputation. Dashed line indicates amputation plane. N=9. Scale bars, 20 µm. (**H**) Quantification of scar score from experiments in (**G**). The scar score is categorized as follows: 1=hearts without persisting collagenous scar; 2=hearts with scar remnants that were covered by a new myocardium; 3=the absence of a new myocardium around the wound. The chi‐squared test was performed. All data are representative of three independent experiments.

Our data indicated that primordial CMs play essential roles during heart development. Next, we examined their contribution to heart regeneration. To address the role of primordial cells during heart regeneration, we perform the below experiments using standardized Mtz treatment protocol (see Methods), with identical treatment conditions applied to adult zebrafish (4–6 months post-fertilization). The *gata4* expression is induced in regenerating CMs and represents the proliferating CMs with reactivation of a cardiac developmental program (Gupta et al., 2013; Kikuchi et al., 2010; Sun et al., 2022b). To determine whether ablation of primordial CMs is sufficient to activate regenerative signaling, we first treated adult *phlda2:mCherry-NTR;gata4:EGFP* fish and control *gata4:EGFP* siblings with Mtz for 12 hr per day over 3 consecutive days without ventricular resection. We did not observe any induction of gata4:EGFP expression following primordial CM ablation (Figure 4—figure supplement 1), indicating that loss of *phlda2*^+^ CMs is not sufficient to trigger regenerative gata4 activation in the absence of injury. Then we treated adult *phlda2:mCherry-NTR;gata4:EGFP* fish and control *gata4:EGFP* siblings with Mtz for 12 hr per day over 3 consecutive days, starting 6 days before resection of the ventricular apex, and collected hearts at 7 dpa. We imaged and quantified *gata4*:EGFP^+^ signals at the injury site and didn’t detect significant difference in EGFP^+^ signals between *phlda2***^+^** cell-depleted fish and control siblings (Figure 4A and B). In addition, with *cmlc2:EGFP* animals, we found that ablation of primordial CMs did not affect the regeneration index score. Myocardial regeneration proceeded normally, with new muscle effectively replacing the injured tissue by 30 dpa (Figure 4C and D). To further assess the requirement of primordial CMs for coronary revascularization, we depleted primordial cells in *phlda2:NTR;deltaC:EGFP* fish and didn’t observe any obvious difference in coronary vessel density in the regenerating area of control siblings (Figure 4E and F). Previous studies have shown that zebrafish hearts can fully regenerate without remaining scars by 30 dpa. We also performed AFOG staining, which labels intact muscle in orange, collagen in blue, and fibrin in red (Poss et al., 2002; Sun et al., 2024). Results showed minimal scar area in primordial cell-depleted hearts, with no obvious difference compared to sibling controls at 30 dpa (Figure 4G and H). Overall, these findings demonstrate that primordial CMs are not essential for heart regeneration, despite their critical role in development.

**Figure 4—figure supplement 1.**
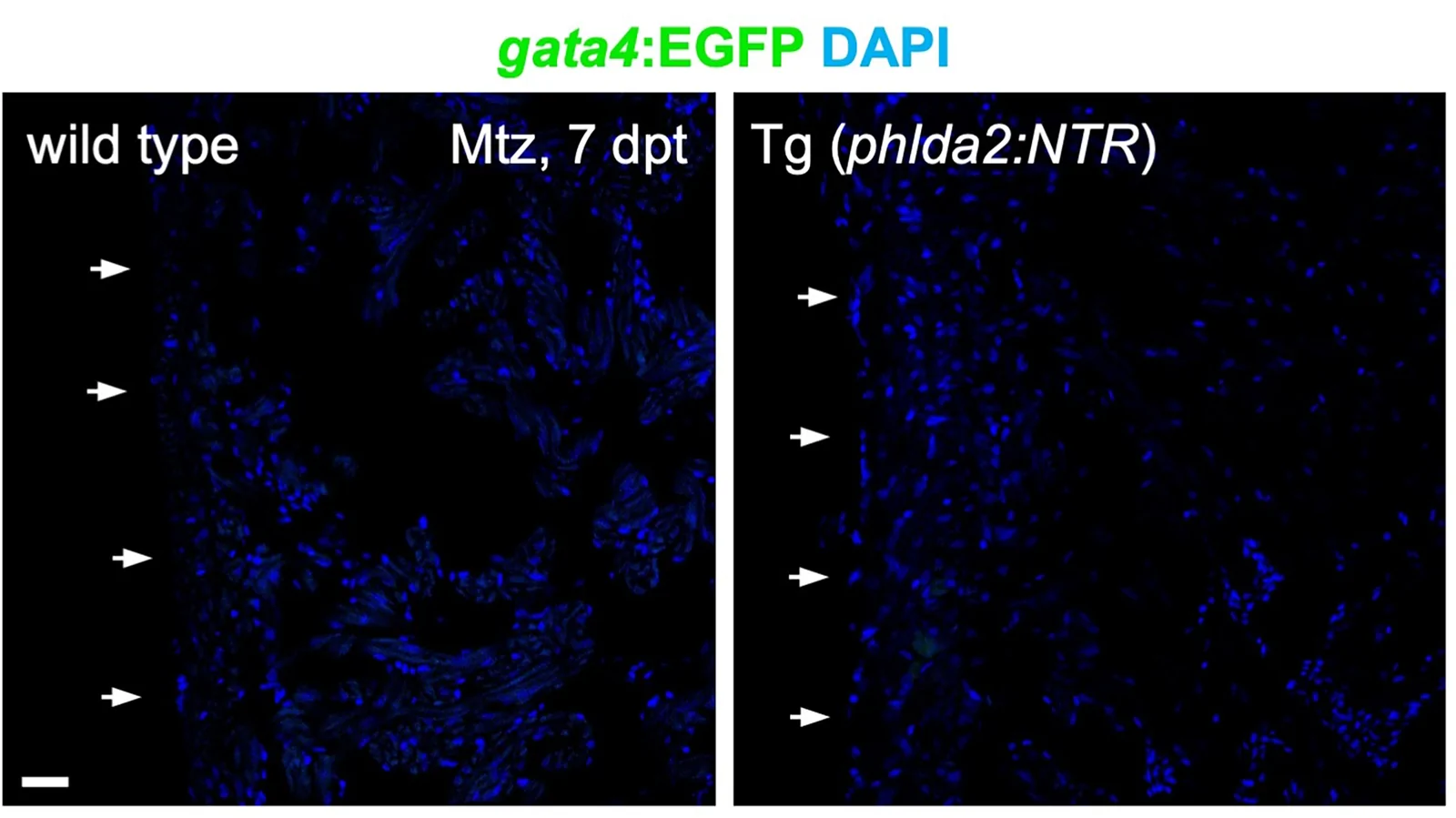
Visualization of *gata4*^+^ cardiomyocytes in sectioned adult *gata4:EGFP* and *gata4:EGFP;phlda2:mCherry-NTR* hearts after Mtz treatment. n=6. Scale bars, 20 µm. All data are representative of two independent experiments.

### Primordial CMs exhibit limited regenerative capacity

**Figure 5.**
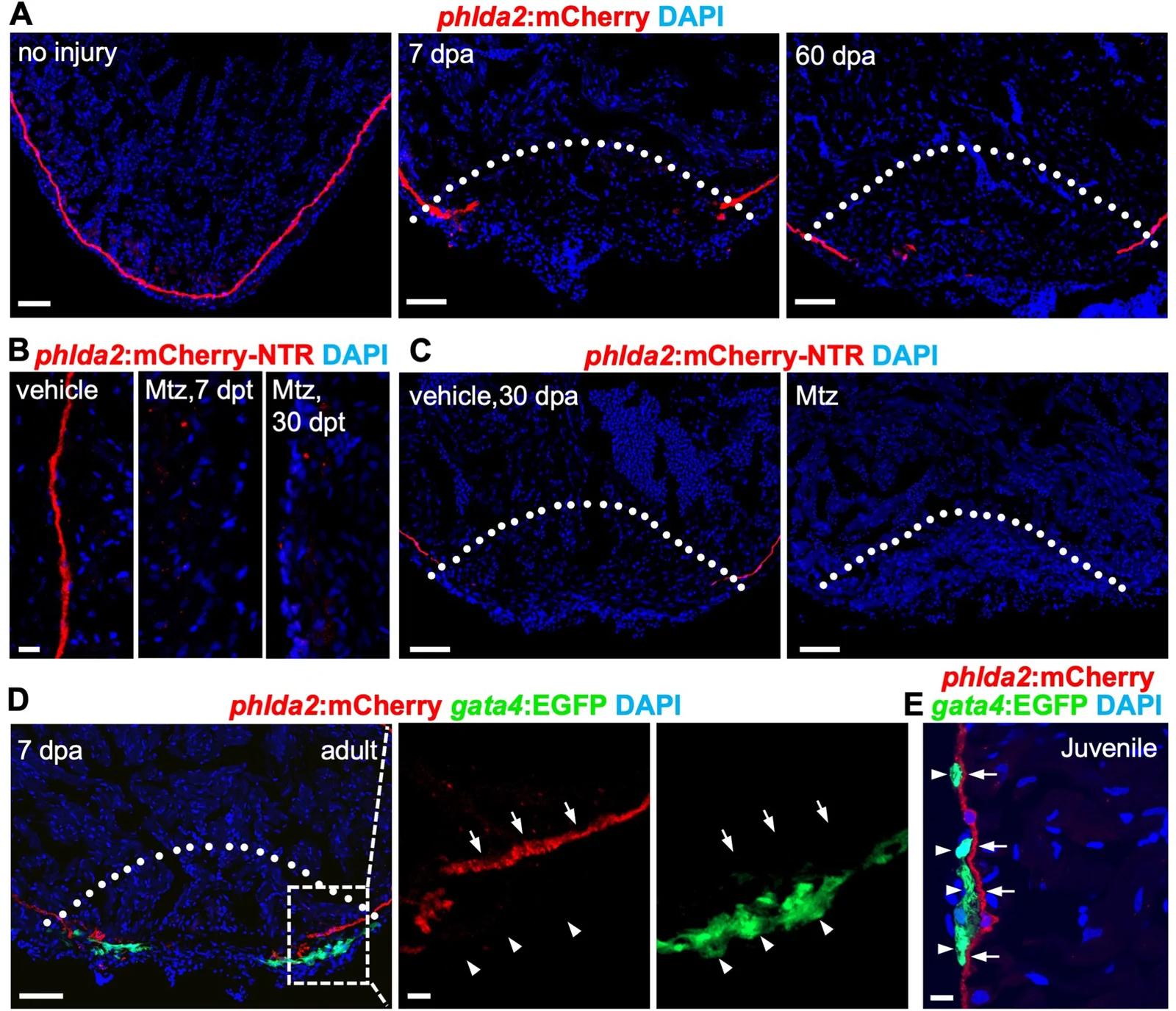
Primordial cardiomyocytes (CMs) have limited regenerative capacity. (**A**) Section images of *phlda2:mCherry-NTR* ventricles without injury and during regeneration at 7 and 60 days post-amputation (dpa). n=11–13. Dashed line indicates amputation plane. Scale bar, 20 μm. (**B**) Section images of *phlda2:mCherry-NTR* ventricles without Mtz treatment and after 7 or 30 days of Mtz treatment. n=12–15. Scale bar, 20 μm. (**C**) Section images of ventricles of vehicle- or Mtz-treated *phlda2a:mCherry-NTR* at 30 dpa. Animals were treated with Mtz during the juvenile stage and amputated as adults. Dashed line indicates amputation plane. n=12. Scale bars, 20 µm. (**D**) Section images of *phlda2:mCherry-NTR;gata4:EGFP* ventricles at 7 dpa assessed for *phlda2*^+^ cells and *gata4*:EGFP^+^ CMs in the injury site. Box area is shown in higher magnification. Arrows represent *phlda2*^+^ cells; arrowheads represent *gata4*^+^ cells. Dashed line indicates amputation plane. n=12. Scale bar, 20 µm. (**E**) Section images of juvenile *phlda2:mCherry-NTR;gata4:EGFP* ventricles assessed for *phlda2*^+^ cells and *gata4*:EGFP^+^ CMs. Arrows represent *phlda2*^+^ cells; arrowheads represent *gata4*^+^ cells. n=6. Scale bar, 10 µm. All data are representative of two independent experiments.

Previous studies have indicated that in adult zebrafish heart, existing CMs can robustly regenerate within several weeks after resection surgery or genetic ablation (Wang et al., 2011). We next assessed the regeneration capacity of primordial CMs. We first examined the *phlda2:mCherry-NTR* hearts at various time points after partial resection of the ventricular apex. The primordial layers were lost at the injury site, even 60 dpa, at which time the heart regeneration was complete (Figure 5A). We next asked whether this limited regenerative capacity reflects an intrinsic limitation of the cells themselves. To test it, we treated the adult *phlda2:mCherry-NTR* animals without resection with Mtz to ablate primordial CMs. We didn’t observe the recovery of *phlda2:*mCherry fluorescence, indicating the failure of primordial regeneration (Figure 5B). Similarly, when juvenile zebrafish (7–8 wpf) were subjected to Mtz-mediated ablation and analyzed 90 dpt, *phlda2*^+^ cells remained absent, demonstrating a persistent failure of primordial CM recovery (Figure 5—figure supplement 1). Since primordial CMs did not regenerate after either apical resection or direct ablation, we therefore asked whether a regenerative cue could overcome this limitation. We performed apical resection after the ablation of primordial CMs and found that primordial CMs still failed to regenerate (Figure 5C), suggesting that their limited regenerative capacity is intrinsic rather than environment dependent. Finally, we found that *phlda2*:EGFP^+^ cells in the injury site did not colocalize with *gata4*:EGFP^+^ cells, suggesting that they have limited proliferative capacity and do not contribute to the new muscle (Figure 5D). Consistently, no overlap between *phlda2*^+^ and *gata4*^+^ CMs was observed in the 7–8 wpf juvenile zebrafish heart (Figure 5E). We conclude that the primordial CMs have intrinsically limited regenerative potential, explaining its absence and functional dispensability in the regenerated heart.

**Figure 5—figure supplement 1.**
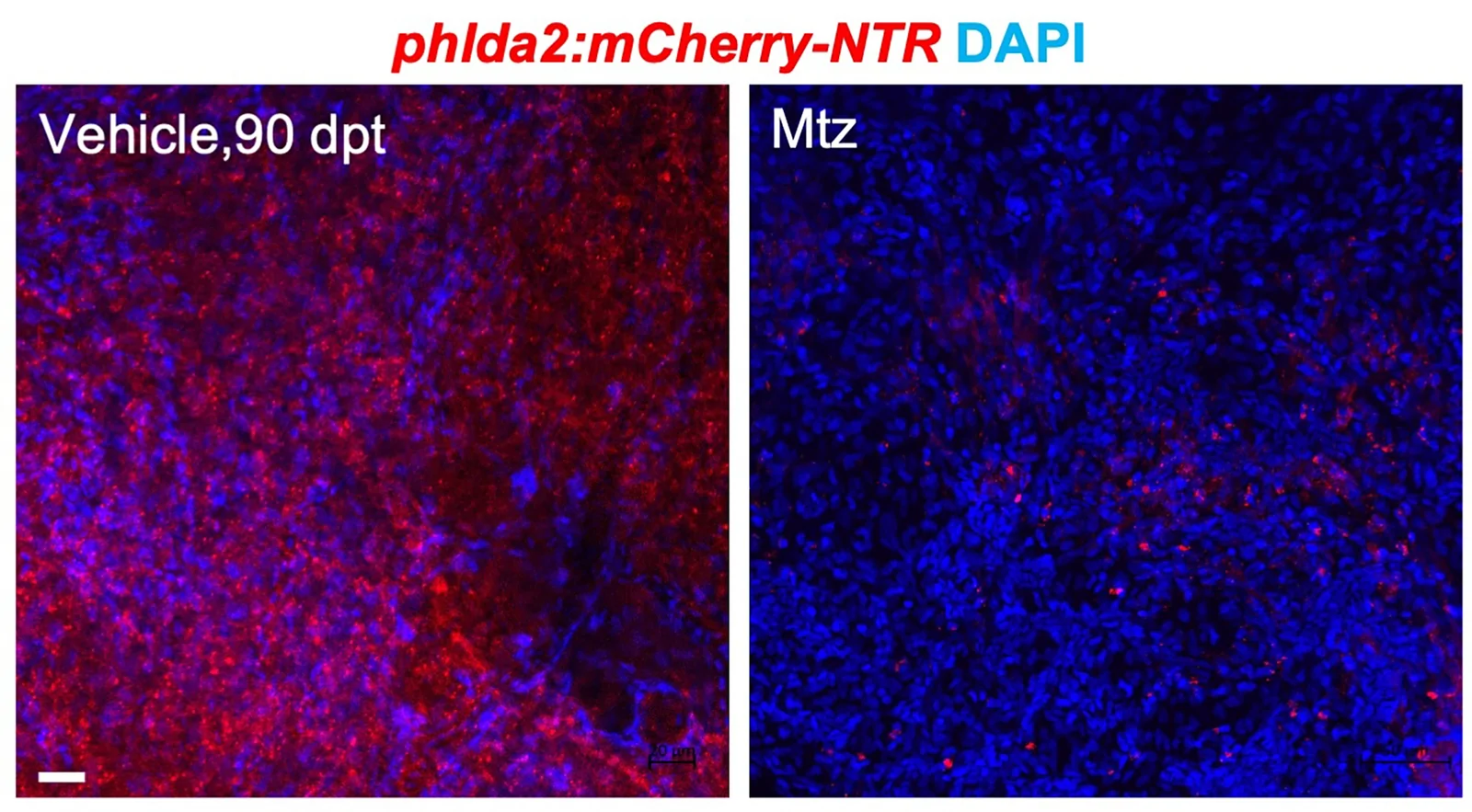
Visualization of *phlda2*^+^ cells in whole-mounted adult *phlda2:mCherry-NTR* hearts after vehicle or Mtz treatment. n=5. Scale bars, 20 µm. All data are representative of two independent experiments.

## Discussion

In this study, we characterized CM subpopulations in the adult zebrafish heart using scRNA-seq and identified *phlda2* as a marker of primordial CMs, which exhibit transcriptional features of an immature cellular state and functions associated with morphogenesis of an epithelial sheet. Using newly generated *phlda2* reporter and ablation lines, we revealed that primordial CMs reside beneath the compact layer and play essential roles in ventricular morphogenesis and coronary vascularization. However, unlike other CM subtypes, these cells display very limited regenerative capacity and are dispensable for myocardial regeneration. Our findings identified primordial CMs as a crucial organizer for heart morphogenesis but found that they play no role during regeneration, revealing a difference between developmental and regenerative programs in the vertebrate heart.

During heart development, multicolor clonal analyses have revealed that the primordial CM lineage contributes to trabecular formation, and trabecular CMs later breach this layer to form the compact myocardium (Gupta and Poss, 2012; Priya et al., 2020; Staudt et al., 2014). However, direct functional evidence has been lacking due to the absence of specific genetic tools. In this study, we generated a *phlda2:mCherry-NTR* line to specifically deplete primordial CMs. The results show that the depletion of these cells caused a marked reduction in the outer trabecular muscle, whereas inner trabeculae remained largely intact, thereby supporting previous reports that primordial CMs, positioned outside the trabecular layer, contribute to the formation of the trabeculae (Gupta and Poss, 2012). Furthermore, we observed a disorganized compact myocardium and disrupted *gata4* expression after the loss of primordial CMs, indicating a crucial role for primordial CMs in coordinating myocardial structural organization and maturation. The loss of these cells likely disrupts the structural and molecular cues required for compact muscle maturation, resulting in an immature ventricular wall.

Coronary vessel formation was also severely affected by the loss of primordial CMs, leading to fragmented and poorly developed vasculature. The coronary vasculature normally invades the compact myocardium during post-embryonic development, a process tightly coupled with myocardial maturation (Harrison et al., 2015; Marín-Juez et al., 2016). Thus, the vascular defects we observed could arise indirectly from the disorganization of the compact myocardium, which fails to provide a proper scaffold for vessel growth. Alternatively, primordial CMs themselves may produce paracrine factors that promote vessel growth and stability. Consistently, our scRNA-seq analysis revealed that *vegfaa* was highly expressed in primordial CMs (Figure 1—figure supplement 1E). VEGF signaling plays a pivotal role in vascular development, acting as an important endothelial mitogen that stimulates endothelial cell tubulogenesis and maintains vessel stability and integrity (Coultas et al., 2005; Darland and D’Amore, 1999; Harrison et al., 2019). These findings suggest that primordial CMs may serve as a critical architectural and signaling hub to coordinate the morphogenesis of both myocardial and vascular structures during heart development. Although primordial CM ablation does not affect survival under laboratory conditions, the observed structural defects may have functional consequences on cardiac performance and overall physiological fitness, which warrant further investigation.

In contrast to their essential role in heart development, primordial CMs appear to play no role in cardiac regeneration. Depletion of these cells did not affect myocardial or vascular regeneration, nor did it impair scar resolution. Furthermore, primordial CMs failed to regenerate after either surgical amputation or genetic ablation, even under strong regenerative conditions. Their inability to regenerate in the injured tissue, together with the lack of overlap with *gata4*^+^ proliferative CMs, indicates that these cells possess intrinsically limited proliferative capacity. They are different from trabecular and compact CMs, which efficiently contribute to the formation of new myocardium. This intrinsic restriction likely explains why the loss of primordial CMs does not impair heart regeneration, suggesting that zebrafish cardiac regeneration is primarily driven by other CM subsets with greater regenerative potential.

Our study demonstrates that primordial CMs are essential for heart morphogenesis but dispensable for regeneration. Although a direct corollary of *phlda2*^+^ primordial CMs has not yet been identified in mammals, mammalian hearts contain heterogeneous CM populations with immature states. Whether these populations represent a functional equivalent of zebrafish primordial CMs remains an open question and needs further investigation. These findings emphasize the functional diversity among CM subtypes and broaden our knowledge of CM heterogeneity in the zebrafish heart. Importantly, our results show that heart regeneration does not simply reactivate the developmental programs across all CMs. Instead, regeneration depends on the CM subsets with high proliferative and regenerative capacity, rather than engaging all CMs equally. This work provides new insights into heart development and regeneration and may inspire new ideas to enhance cardiac repair in mammals.

## Methods

### Zebrafish and heart injuries

4- 10-month-old outbred EK or EK/AB zebrafish were used for ventricular resection surgeries. To deplete *phlda2*-expressing cells, *phlda2:mCherry-NTR* transgenic zebrafish were used at the age of 6–8 weeks (juvenile) and 4–12 months (adult). Animal density was maintained at ~4 fish/L in all experiments. The *phlda2:mCherry-NTR* animals and control wild-type siblings were treated with vehicle or 10 mM metronidazole (Mtz) (MilliporeSigma) in a 1.5 L mating tank for 12 hr/day to ablate *phlda2*^+^ cells. Transgenic strains described elsewhere include *gata4:EGFP* (Heicklen-Klein and Evans, 2004)*, cmlc2:EGFP* (Kikuchi et al., 2010), *tcf21:mCherry* (Wang et al., 2015), and *deltaC:EGFP* (Sun et al., 2022a). All transgenic strains were analyzed as hemizygotes. All animal procedures were performed in accordance with Emory University guidelines.

### Constructs, zebrafish transgenes

*phlda2:EGFP*. The *phlda2* translational start codon in the BAC clone CH211-193F12 was replaced with *EGFP* by Red/ET recombineering technology (GeneBridges). 5′ and 3′ homologous arms flanking the *EGFP* cassette for recombination were a 248-base-pair (bp) fragment upstream of the start codon and a 364 bp fragment downstream, respectively. BACs for transgenesis were purified with a midi-prepare 100 kit (QIAGEN) and co-injected with PI-SceI into one-cell-stage zebrafish embryos. The full name of this transgenic line is Tg (*phlda2:EGFP*)*^em21^*.

*phlda2:mCherry-NTR*. The *phlda2* translational start codon in the BAC clone CH211-193F12 was replaced with *mCherry-NTR* by Red/ET recombineering. 5′ and 3′ homologous arms flanking the *mCherry-NTR* cassette for recombination were a 248 bp fragment upstream of the start codon, and a 364 bp fragment downstream, respectively. The full name of this transgenic line is Tg (*phlda2:mCherry-NTR*)*^em22^*.

### Mtz treatment

For conditional ablation of *phlda2*^+^ CMs, zebrafish expressing *phlda2:mCherry-NTR* were treated with 10 mM Mtz for 12 hr per day for 3 consecutive days. Fish were washed out and maintained in fresh system water after each daily treatment. For developmental analyses, juvenile zebrafish (7–8 wpf) were subjected to Mtz treatment as described above. Following completion of Mtz exposure, fish were maintained under standard conditions. Hearts were collected at 10 dpt for assessment of gata4 activation, and at 30 dpt for analysis of myocardial structure and coronary vessel development. For regeneration experiments, adult zebrafish (4–6 months of age) were similarly treated with Mtz for 3 consecutive days with daily washout. Three days after the final Mtz treatment, ventricular apex resection was performed. Hearts were harvested at 7 days post-amputation (dpa) for analysis of gata4 activation and early regenerative responses, and at 30 dpa for evaluation of myocardial regeneration and coronary vessel revascularization.

### Histological methods and quantification

Histological analyses were performed on 10 µm cryosections or whole-mount paraformaldehyde-fixed hearts. Sectioned ventricular tissues were imaged using a Zeiss LSM800 confocal scanning microscope with ZEN V3.7.F.

To quantify trabecular muscle loss, three medial longitudinal sections were selected from each heart and imaged. Single optical slices of the ventricle were acquired using a 20× objective lens (1024×1024 pixels) at the same ventricular position in sections from *cmlc2:EGFP* hearts. Trabecular muscle loss was quantified as the ratio of *cmlc2*:EGFP^+^ cell area to the total heart area within a region extending 100 μm from the ventricular wall. Quantification of *gata4*:EGFP^+^ expression in adult fish was performed as described (Sun et al., 2022b).

To quantify coronary expansion in developing hearts from *deltaC:EGFP* fish, whole-mounted specimens were selected, and ventricle images were captured using a 20× objective lens (1024×1024 pixels). The coronary vessel lengths were measured using ImageJ software. To quantify the vessel broken, the total number of vessel fragments were counted. To quantify coronary vessel expansion in regenerating hearts from *deltaC:EGFP* fish, images of ventricles were captured using a 20× objective lens (1024×1024 pixels). The GFP signals were measured in pixels using ImageJ software for signals in the injury site.

To quantify *gata4*:EGFP^+^ expression in developing hearts from *gata4:EGFP* fish, whole-mounted specimens were selected and ventricle images were captured using a 20× objective lens (1024×1024 pixels). The GFP signals were measured in pixels and mean intensity using ImageJ software in the injury site. To quantify *gata4*:EGFP^+^ expression in regenerating hearts from *gata4:EGFP* fish, images of ventricles were captured using a 20× objective lens (1024×1024 pixels). The GFP signals were measured in pixels using ImageJ software for signals in the injury site.

### AFOG staining

A triple staining with Aniline Blue, acid Fuchsin, and Orange-G (AFOG) was performed as previously described (Poss et al., 2002). Briefly, cryosections were fixed in Bouin’s solution (Sigma-Aldrich) for 2 hr at 60°C and 1 hr at room temperature. The slides were washed for 30 min in tap water and then treated with 1% phosphomolybdic acid (Sigma-Aldrich) and 0.25% phosphotungstic acid solution (Sigma-Aldrich) for 5 min. They were rinsed with distilled water, and incubated for 5 min with AFOG solution containing 3 g acid Fuchsin (Sigma-Aldrich), 2 g Orange G (Sigma-Aldrich), 1 g Aniline Blue (Sigma-Aldrich) in distilled water, with pH adjusted to 1.1. After 5 min wash with distilled water, the sections were dehydrated in graded series of ethanol and two changes of xylene, then mounted with Permount mounting medium (Fisher Chemical). Degree of regeneration was scored in a blind-test assessment of AFOG-stained sections at 30 dpa. Three sections with evident injury were selected per heart. The criteria for scoring were based on the amount of persisting collagen (blue staining) and the wound closure with a new myocardium. The hearts without persisting collagenous scar were classified as fully regenerated hearts. Hearts with scar remnants that were covered by a new myocardium were considered as incomplete regeneration. The absence of a new myocardium around the wound was considered as a blocked regeneration.

### Single-cell RNA sequencing

To prepare *cmlc2*^+^ cells for scRNA-seq analyses, *cmlc2:EGFP* transgenic fish were raised to adult stages. Adult hearts were collected at 4 months of age. The heart samples were digested with 0.26 U/mL Liberase Thermolysin Medium (TM) based on a previously published protocol (Spanjaard et al., 2018). Dissociated cells were spun down and live EGFP^+^ cells were sorted by flow cytometry. Isolated cells were sent to Emory Integrated Genomics Core (EIGC) center for 10× scRNA-seq. scRNA-seq libraries were prepared using the Chromium Single Cell 3’ Library & Cell Bead Kit v3.1 (Cat. No. 1000128, 1000127, 120262; 10× Genomics) according to the manufacturer’s protocol. Libraries were sequenced with an Illumina NextSeq550 using mid-output 150-cycle kits according to the manufacturer’s specifications. The newly generated scRNA-seq data were demultiplexed, aligned, and quantified using Cell Ranger Single-Cell Software. The newly generated scRNA-seq data yielded 136,174,297 total reads with an average sequencing depth of 36,168 reads per cell. Preliminary filtered data generated from Cell Ranger were used for downstream analysis by the Seurat R package according to standard workflow.

The scRNA-seq dataset has been submitted to Gene Expression Omnibus (GEO). To review, please use the GEO accession number GSE312374 with the token gbulwsiivdqlven.

### Statistical analysis

All data are presented as mean ± standard error of the mean (SEM). All statistical analyses were performed using GraphPad Prism 7 software. The Mann-Whitney rank-sum test was used for assessing statistical differences between the two groups. Results with p-values<0.05 were considered statistically significant. Statistical details of experiments can be found in the figures and figure legends.

## Data Availability

The scRNA-seq dataset has been submitted to Gene Expression Omnibus (GEO). Accession number GSE312374. The following dataset was generated: SunJ ChenL WangJ 2025Primordial cardiomyocytes orchestrate myocardial morphogenesis and vascularization but are dispensable for regenerationNCBI Gene Expression OmnibusGSE31237410.7554/eLife.110256PMC1355306742709570

## Additional files

Supplementary file 1. Top differentially expressed genes for each cardiomyocyte (CM) cluster.

Supplementary file 2. Gene Ontology (GO) term enrichment results.

MDAR checklist
