# Supplementary material for: Primordial cardiomyocytes orchestrate myocardial morphogenesis and vascularization but are dispensable for regeneration
Source: eLife. 2026 Sep 8;15:RP110256. doi: 10.7554/eLife.110256 (PMC13553067; doi:10.7554/eLife.110256)
Supplement: Peer review history

# Editor report: eLife Assessment

**DOI:** 10.7554/eLife.110256.3.sa0

**Authors:** Juan-Manuel González-Rosa

**Affiliations:** Boston College United States

**Keywords:** Important, Solid

This **important** work identifies phlda2 as a specific marker for primordial cardiomyocytes in the adult zebrafish heart and demonstrates their essential role in myocardial morphogenesis and coronary vascularization, but not in heart regeneration. The conclusions are well supported by single-cell transcriptomics, new genetic tools, and cell-specific ablation experiments. The revised version strengthens these findings with analyses of the epicardium, long-term time points showing that the developmental defects persist, and a control confirming that loss of primordial cardiomyocytes alone does not trigger a regenerative response. Overall, the evidence is **solid** and provides insight into the difference between developmental and regenerative cardiac programs, and this work will be of interest for those studying cardiac development and regeneration.

# Referee report: Reviewer #1 (Public review)

**DOI:** 10.7554/eLife.110256.3.sa1

Summary:

This manuscript addresses an important question in cardiac biology: whether distinct cardiomyocyte (CM) subpopulations play specialized roles during heart development and regeneration. Using single-cell RNA sequencing and newly generated genetic tools, the authors identify phlda2 as a specific marker of primordial cardiomyocytes in the adult zebrafish heart. They further show that these primordial CMs function are essential for myocardial morphogenesis and coronary vascularization but are dispensable for myocardial regeneration or revascularization after injury. These findings indicate that heart regeneration doesn't simply recapitulate developmental processes.

Strengths:

A major strength of the study is the generation of a phlda2 BAC reporter, which provides a specific and reliable marker for primordial cardiomyocytes. The lack of genetic tools has previously limited functional analysis of this CM population. By using phlda2 regulatory elements to generate reporter and NTR-based ablation lines, the authors can visualize and selectively manipulate primordial CMs in vivo. This enables a direct functional interrogation rather than relying on lineage tracing or correlative evidence. Through genetic ablation, the authors convincingly demonstrate that primordial CMs are essential for myocardial morphogenesis and coronary vascular organization during development but are not necessary for heart regeneration.

Weaknesses:

(1) The manuscript would benefit from clarifying whether the primordial cardiomyocytes ablation affects epicardial cell behaviors during heart development, given that the well-established role of the epicardium in supporting coronary vessel growth, it is possible that the vascular phenotypes observed after primordial CM ablation may be affected, at least in part, by altered epicardial cells.

(2) Because primordial cardiomyocytes form a dense, single-cell-thick layer covering the ventricular surface, it would be informative to determine whether their loss alters the spatial distribution or inward migration of coronary endothelial cells or epicardial cells.

(3) The manuscript carefully examines the relationship between primordial CMs and gata4⁺ cardiomyocytes during regeneration. However, their relationship during heart development should be more fully addressed.

(4) As loss of cardiomyocytes is known to induce gata4:GFP activation during regeneration, it would be important to determine whether ablation of primordial cardiomyocytes alone triggers gata4:GFP expression in neighboring cardiomyocytes. This analysis would further support the conclusion that primordial cardiomyocytes are not required for regenerative responses.

# Referee report: Reviewer #2 (Public review)

**DOI:** 10.7554/eLife.110256.3.sa2

Summary:

In the manuscript "Primordial Cardiomyocytes orchestrate myocardial morphogenesis and vascularization but are dispensable for regeneration", Sun et al. identify a novel marker of primordial cardiomyocytes and use it to visualize and ablate the population during development and regeneration. The role of the primordial layer has not been investigated because the tools to manipulate this population have not existed. The manuscript is straightforward, easy to understand, and addresses an important question that has not been explored.

While the manuscript provides important insights into the role of primordial CMs, backed by a convincing methodology, the authors should clarify their requirements for heart development and maturation. Specifically, is the primordial layer required for the fish to survive? Do primordial CMs regenerate when ablated during development, and do the defects observed (in trabecular and compact CMs and coronary vessels) resolve after 10 days post-treatment when they were detected?

Strengths:

The major strengths are the identification of a marker that enables manipulation of primordial cardiomyocytes and the tools generated by the team.

Weaknesses:

The major weakness is not considering the longer-term consequences of primordial layer ablation during development, as it is unclear whether the animals succumb to the acute cardiac defects observed or fully recover.

# Referee report: Reviewer #3 (Public review)

**DOI:** 10.7554/eLife.110256.3.sa3

Summary:

The authors performed single-cell RNA sequencing of adult zebrafish hearts and identified markers for distinct cardiomyocyte subpopulations. One marker, phlda2, marks primordial cardiomyocytes. They generated transgenic reporter lines to characterize phlda2 expression patterns and a phlda2-NTR ablation line to determine the functional requirement of primordial cardiomyocytes during heart regeneration. They found that phlda2+ primordial cardiomyocytes are essential for myocardial morphogenesis and coronary vessel development. Interestingly, when phlda2+ primordial cardiomyocytes are ablated during heart regeneration, gata4+ cortical cardiomyocytes, coronary vessel revascularization, and scar tissue formation are not affected.

Strengths:

The authors identified a new primordial cardiomyocyte marker, phlda2. They further demonstrated that primordial cardiomyocytes are important for heart morphogenesis but dispensable for heart regeneration. Their findings reveal a potential difference between heart development and regeneration programs.

Weakness:

Despite the interesting findings, the authors did not provide supplemental data for their scRNAseq to demonstrate the data quality and support their conclusions, and some results are not well described.

# Author comment: Author response

**DOI:** 10.7554/eLife.110256.3.sa4

**Authors:** Jisheng Sun, Lu Chen, Jinhu Wang

**Affiliations:** Fudan University Shanghai China; Emory University School of Medicine Atlanta United States; Emory University School of Medicine Atlanta United States

The following is the authors’ response to the original reviews.

**Public Reviews:**

**Reviewer #1 (Public review):**

Summary:

This manuscript addresses an important question in cardiac biology: whether distinct cardiomyocyte (CM) subpopulations play specialized roles during heart development and regeneration. Using single-cell RNA sequencing and newly generated genetic tools, the authors identify phlda2 as a specific marker of primordial cardiomyocytes in the adult zebrafish heart. They further show that these primordial CMs function are essential for myocardial morphogenesis and coronary vascularization but are dispensable for myocardial regeneration or revascularization after injury. These findings indicate that heart regeneration doesn't simply recapitulate developmental processes.

Strengths:

A major strength of the study is the generation of a phlda2 BAC reporter, which provides a specific and reliable marker for primordial cardiomyocytes. The lack of genetic tools has previously limited functional analysis of this CM population. By using phlda2 regulatory elements to generate reporter and NTR-based ablation lines, the authors can visualize and selectively manipulate primordial CMs in vivo. This enables a direct functional interrogation rather than relying on lineage tracing or correlative evidence. Through genetic ablation, the authors convincingly demonstrate that primordial CMs are essential for myocardial morphogenesis and coronary vascular organization during development but are not necessary for heart regeneration.

Weaknesses:

(1) The manuscript would benefit from clarifying whether the primordial cardiomyocytes ablation affects epicardial cell behaviors during heart development, given that the wellestablished role of the epicardium in supporting coronary vessel growth, it is possible that the vascular phenotypes observed after primordial CM ablation may be affected, at least in part, by altered epicardial cells.

We thank the reviewer for this important suggestion. To address this possibility, we examined epicardial cells in primordial CM-ablated hearts using the epicardial marker tcf21. Surprisingly, we found that epicardial cells rapidly expanded following primordial CM ablation, with an increase already detected at 5 days post-treatment. This increase persisted through at least 30 dpt, when epicardial cell abundance remained higher than that in control hearts. These findings suggest that the coronary vessel defects are unlikely to result from a reduction in epicardial cell number. In addition, we investigated whether loss of the primordial CM layer permits abnormal inward migration of epicardial cells or coronary vessels into the myocardium. However, we observed no evidence of ectopic localization of either cell population following primordial CM ablation. While we cannot exclude the possibility that altered epicardial function or signaling contributes to the vascular phenotype, our results indicate that the vascular defects are not attributable to reduced epicardial cell abundance or abnormal epicardial invasion. We have included these experiments in Page 10 and 11, and Fig. 3I-3J of revised manuscript.

“Because epicardial cells play critical roles in coronary vessel development, we next asked whether the vascular defects observed following primordial CM ablation were secondary to alterations in the epicardium. We found that tcf21+ epicardial cells revealed an increase rather than a decrease in epicardial cell abundance in primordial CM-ablated hearts (Fig. 3I3J). These findings suggest that the impaired coronary vessel organization is unlikely to result from a reduction in epicardial cell number, although we cannot exclude the possibility that altered epicardial function or signaling contributes to the vascular phenotype.”

(2) Because primordial cardiomyocytes form a dense, single-cell-thick layer covering the ventricular surface, it would be informative to determine whether their loss alters the spatial distribution or inward migration of coronary endothelial cells or epicardial cells.

We appreciate the reviewer for this insightful suggestion. Because primordial cardiomyocytes form a continuous single-cell-thick layer at the ventricular surface, we examined whether their ablation affects the spatial distribution or promotes inward migration of epicardial cells or coronary endothelial cells. Using tcf21 and deltaC reporters, we carefully analyzed the localization of these cell populations within the myocardium. We did not observe any evidence of abnormal inward migration or ectopic localization of either epicardial cells or coronary endothelial cells following primordial CM ablation (Fig. 3J–3K). These results indicate that loss of the primordial CM layer does not disrupt tissue compartmentalization or lead to inappropriate cellular invasion into the myocardial interior. We have included these experiments in Page 11, and Fig. 3J-3K of revised manuscript.

“In addition, because primordial cardiomyocytes form a continuous layer at the ventricular surface, we investigated whether their ablation permits abnormal invasion of epicardial cells or coronary vessels into the myocardium. However, we observed no evidence of ectopic localization of either cell population following primordial CM ablation (Fig. 3J-3K). Thus, loss of the primordial CM layer does not appear to disrupt tissue compartmentalization or permit abnormal cellular invasion into the myocardium.”

(3) The manuscript carefully examines the relationship between primordial CMs and gata4^+^ cardiomyocytes during regeneration. However, their relationship during heart development should be more fully addressed.

We thank the reviewer for this important suggestion. To further address the relationship between primordial cardiomyocytes and *gata4*^+^ cardiomyocytes during heart development, we examined their spatial and cellular relationship in juvenile zebrafish hearts. Consistent with our observations during regeneration, we did not detect any overlap between *phlda2*^+^ cardiomyocytes and *gata4*^+^ cardiomyocytes in the juvenile heart (7-8 wpf). These results indicate that primordial CMs and *gata4*^+^ proliferative CMs represent distinct cardiomyocyte populations during both heart development and regeneration. We have included these experiments in Page 13, and Fig.5E of revised manuscript.

“Consistently, no overlap between *phlda2*^+^ and *gata4*^+^ cardiomyocytes was observed in the wpf juvenile zebrafish heart (Fig. 5E).”

(4) As loss of cardiomyocytes is known to induce gata4:GFP activation during regeneration, it would be important to determine whether ablation of primordial cardiomyocytes alone triggers gata4:GFP expression in neighboring cardiomyocytes. This analysis would further support the conclusion that primordial cardiomyocytes are not required for regenerative responses.

We appreciate the reviewer for this important suggestion. To determine whether ablation of primordial cardiomyocytes alone is sufficient to activate regenerative signaling, we treated adult *phlda2:mCherry-NTR;gata4:EGFP* fish and gata4:EGFP siblings with Mtz for 12 hours per day over three consecutive days without ventricular resection. Under these conditions, we did not observe any induction of gata4 expression following primordial CM ablation (Fig. S5). These results indicate that loss of *phlda2*^+^ cardiomyocytes alone is not sufficient to trigger regenerative gata4 activation in the absence of injury, further supporting that primordial CMs are dispensable for activation of the regenerative response. We have included these experiments in Page 11 and Fig. S5 of revised manuscript.

“To determine whether ablation of primordial cardiomyocytes is sufficient to activate regenerative signaling, we first treated adult *phlda2:mCherry-NTR;gata4:EGFP* fish and control *gata4:EGFP* siblings with Mtz for 12 hours per day over three consecutive days without ventricular resection. We did not observe any induction of *gata4:EGFP* expression following primordial CM ablation (Fig. S5), indicating that loss of *phlda2*^+^ cardiomyocytes is not sufficient to trigger regenerative gata4 activation in the absence of injury.”

**Reviewer #2 (Public review):**

Summary:

In the manuscript "Primordial Cardiomyocytes orchestrate myocardial morphogenesis and vascularization but are dispensable for regeneration", Sun et al. identify a novel marker of primordial cardiomyocytes and use it to visualize and ablate the population during development and regeneration. The role of the primordial layer has not been investigated because the tools to manipulate this population have not existed. The manuscript is straightforward, easy to understand, and addresses an important question that has not been explored.

While the manuscript provides important insights into the role of primordial CMs, backed by a convincing methodology, the authors should clarify their requirements for heart development and maturation. Specifically, is the primordial layer required for the fish to survive?

We thank the reviewer for this important question. We found that efficient ablation of *phlda2*^+^ primordial cardiomyocytes does not affect overall survival of zebrafish under standard laboratory conditions. Although these animals exhibit clear defects in cardiac structure and coronary vascular organization, they remain viable during the experimental period, indicating that the primordial CM layer is not essential for survival. While we did not assess detailed physiological parameters such as cardiac function, swimming behavior, or long-term fitness in this study, the observed structural abnormalities suggest that subtle functional consequences may exist. These aspects will be important directions for future investigation. We have included the description on page 14, paragraph 2.

“Although primordial CM ablation does not affect survival under laboratory conditions, the observed structural defects may have functional consequences on cardiac performance and overall physiological fitness, which warrant further investigation.”

Do primordial CMs regenerate when ablated during development, and do the defects observed (in trabecular and compact CMs and coronary vessels) resolve after 10 days posttreatment when they were detected?

We appreciate the reviewer for this important question. To determine whether primordial cardiomyocytes regenerate following ablation during development, we performed Mtz-mediated ablation in juvenile zebrafish (7–8 wpf) and examined the hearts at extended time points after treatment. We found that *phlda2*^+^ cardiomyocytes did not recover even at 90 days post-treatment, indicating a persistent loss of this population following developmental-stage ablation (Fig. S6). Importantly, we further assessed whether the cardiac defects observed at earlier time points resolve over time. We found that the abnormalities in trabecular and compact myocardium, as well as coronary vessel organization, persisted at 90 days post-treatment and did not show evidence of recovery (Fig. S4C). These findings demonstrate that the observed defects are not transient developmental delays but represent long-lasting structural alterations of the heart following primordial CM ablation.

Major Comments:

(1) Figure 1: A more detailed characterization of the three CM populations would be helpful in the text as well as a new Supplemental Excel Data Sheet with the top unique genes expressed in each.

We thank the reviewer for this helpful suggestion. In the revised manuscript, we have expanded the description of the three cardiomyocyte populations in the Results section to provide a more detailed characterization. We have revised the description on page 8. In addition, we have added a new Supplemental Excel Data Sheet (Table S1) listing the top differentially expressed genes for each CM cluster.

“Notably, Cluster 2 showed additional enrichment for pathways involved in mitochondrial respiratory chain assembly, ATP synthesis, TCA cycle, and ribosome biogenesis, suggesting a relatively higher metabolic and biosynthetic activity state. In contrast, Cluster 1 was enriched for GO terms associated with mitochondrial stress responses, protein degradation, and cytoprotective pathways, indicating a stress-adapted cardiomyocyte state. Cluster 3 showed reduced enrichment of metabolic pathways, consistent with an immature metabolic profile, and was further enriched for genes involved in muscle development and epithelial morphogenesis, suggesting a role in cardiac morphogenesis and tissue organization.”

(2) Figure 3: Lower magnification views of control and MTZ-treated hearts are needed for all reporters shown (cmlc2, gata4, deltaC) at 10 days post-treatment. These "whole heart" views will enable the reader to get a gross sense of how disrupted heart development is following ablation of the primordial layer.

We appreciate the reviewer for this helpful suggestion. We have added low-magnification whole-heart images for all reported markers (*cmlc2*, *gata4*, and *deltaC*) to better illustrate the overall cardiac morphology following ablation of the primordial layer. We have included these data in Fig. S3 of revised manuscript.

(3) It should also be stated clearly in the Results section text what day post-fertilization Mtx treatment began. A section on Mtx treatment, including timing (what day was it applied and what day was it washed out) and dose, should be added to the methods section.

We thank the reviewer for this helpful suggestion. In response, we have now clearly stated the timing of Mtz treatment in the Results section in page 9, 10 and 11 of revised manuscript.

“To address the role of *phlda2*^+^ cells during heart development, we performed the following experiments using a standardized Mtz treatment protocol (see Methods), with identical treatment conditions applied to 7-8 wpf juvenile zebrafish.”

“To address the role of primordial cells during heart regeneration, we perform the below experiments using standardized Mtz treatment protocol (see Methods), with identical treatment conditions applied to adult zebrafish (4–6 months post-fertilization).”

In addition, we have added a Mtz treatment section in the Methods, which now includes detailed information on dosage, duration, and washout schedule to ensure full reproducibility of the experiments.

“Mtz treatment

For conditional ablation of *phlda2*^+^ cardiomyocytes, zebrafish expressing *phlda2:mCherryNTR* were treated with 10 mM metronidazole (Mtz) for 12 hours per day for three consecutive days. Fish were washed out and maintained in fresh system water after each daily treatment. For developmental analyses, juvenile zebrafish (7–8 weeks post-fertilization) were subjected to Mtz treatment as described above. Following completion of Mtz exposure, fish were maintained under standard conditions. Hearts were collected at 10 days post-treatment for assessment of gata4 activation, and at 30 days post-treatment for analysis of myocardial structure and coronary vessel development. For regeneration experiments, adult zebrafish (4–6 months old) were similarly treated with Mtz for three consecutive days with daily washout. Three days after the final Mtz treatment, ventricular apex resection was performed. Hearts were harvested at 7 days post-amputation (dpa) for analysis of gata4 activation and early regenerative responses, and at 30 dpa for evaluation of myocardial regeneration and coronary vessel revascularization.”

(4) What happens to the heart 2 and 6 months post-treatment? Are there long-term consequences to primordial layer ablation or do the defects seen at 10 days post-treatment eventually resolve?

We thank the reviewer for this important question. To determine whether the developmental defects observed following primordial CM ablation are transient or persist long term, we performed additional analyses at later time points after Mtz washout. First, we found that *phlda2*^+^ cardiomyocytes failed to recover following Mtz-mediated ablation. In adult *phlda2:mCherry-NTR* fish, phlda2^+^ cells remained absent 30 days after Mtz washout (Fig. 5B). Similarly, when juvenile fish (7–8 wpf) were treated with Mtz and subsequently allowed to recover, *phlda2*^+^ cells were still not restored at 90 days post-treatment (Fig. S6). Second, juvenile zebrafish (7–8 wpf) were subjected to Mtz-mediated primordial CM ablation and analyzed 90 days after Mtz washout. We found that vascular abnormalities persisted long after ablation of primordial CMs (Fig. S4C). Coronary vessels remained disorganized and fragmented, indicating that the vascular phenotype does not resolve over time. Together, these findings demonstrate that primordial CM ablation causes long-lasting defects and that the abnormalities observed are not transient developmental delays. Instead, loss of primordial CMs results in persistent cellular and vascular defects that remain evident months after Mtz treatment. We have included these experiments in Fig. S4C and Fig. S6 of revised manuscript.

“Notably, these vascular abnormalities persisted at 90 days post-treatment, indicating that the defects do not resolve during subsequent cardiac growth and maturation (Fig. S4C).”

“Similarly, when juvenile zebrafish (7–8 wpf) were subjected to Mtz-mediated ablation and analyzed 90 days after treatment, *phlda2*^+^ cells remained absent, demonstrating a persistent failure of primordial CM recovery (Fig. S6).”

(5) Also, does the primordial layer come back in these animals where the lineage is ablated during development? Or is it permanently lost as shown in Figure 5 when it is ablated during adulthood?

We appreciate the reviewer for raising this important question. To determine whether the primordial layer can be reestablished following ablation during development, we treated juvenile *phlda2:mCherry-NTR* fish (7–8 wpf) with Mtz and examined hearts 90 days after treatment. We found that *phlda2*^+^ cardiomyocytes remained absent at this late time point (Fig. S6), indicating that the primordial layer does not recover following developmental-stage ablation. We have included these experiments in Page 12, and Fig. S6 of revised manuscript.

“Similarly, when juvenile zebrafish (7–8 wpf) were subjected to Mtz-mediated ablation and analyzed 90 days after treatment, *phlda2*^+^ cells remained absent, demonstrating a persistent failure of primordial CM recovery (Fig. S6).”

(6) Figure 4: Need to show that phlda2 reporter fluorescence in lost/reduced following Mtz treatment during adulthood before apex amputation.

We thank the reviewer for this important suggestion. We agree that confirming efficient ablation of *phlda2*^+^ cardiomyocytes in adult fish prior to regeneration analysis is essential.

In our study, we have already demonstrated in Fig.5B that Mtz treatment in adult *phlda2:mCherry-NTR* fish results in efficient and sustained loss of *phlda2*^+^ cells, with no detectable recovery at 7 and 30 days post-treatment. These data confirm robust ablation of the primordial CM population following Mtz treatment in adults. Therefore, additional redundant imaging prior to apex resection was not performed in Fig 4.

(7) Figure 5: It is interesting that primordial CMs do not regenerate following apex amputation or genetic ablation. This result suggests that primordial CMs are only important during development and dispensable during adulthood? This result also makes me question whether primordial CMs are actually required for heart development, which is why it is important to address whether the fish recovers.

We appreciate the reviewer for this insightful comment. Our data indicate that primordial cardiomyocytes are essential for proper heart development, as their ablation during juvenile stages leads to significant structural and vascular abnormalities. Importantly, we further examined long-term outcomes and found that these defects do not resolve over time. Juvenile zebrafish subjected to primordial CM ablation failed to recover phlda2^+^ cardiomyocytes even at 90 days post-treatment, and coronary vascular abnormalities also persisted at this late stage (Fig. S4C). These findings indicate that the observed developmental defects are not transient delays but instead reflect long-lasting structural alterations of the heart. In addition, we found that adult zebrafish similarly fail to regenerate primordial cardiomyocytes following genetic ablation (Fig. 5B), further supporting the limited regenerative capacity of this population. Together, these data demonstrate that primordial cardiomyocytes are required for proper cardiac development, and their loss leads to persistent defects that are not reversed during subsequent growth or regeneration.

Minor:

Line 234: Did the authors mean to write Cluster 3 (instead of Cluster 2)?

We thank the reviewer for pointing out this error. We confirm that this was a labeling mistake, and “Cluster 3” is correct. The text has been corrected in the revised manuscript.

Line 265: There is a typo of some sort in the phrase, "54.7% reduction closed to the ventricular wall".

We thank the reviewer for pointing out this error. We have changed the description on page 10 of the revised manuscript.

“The compact myocardium was disorganized compared with controls, and trabecular muscle formation was severely impaired, with an approximately 54.7% reduction in trabecular area, predominantly observed in regions adjacent to the ventricular wall (Fig. 3A, 3B and S3A).”

Is there a corollary lineage in mammals? This should be addressed in the Introduction or Discussion.

We thank the reviewer for this insightful suggestion. At present, a direct corollary lineage to zebrafish *phlda2*^+^ primordial cardiomyocytes have not been clearly defined in mammals. However, mammalian hearts also contain heterogeneous cardiomyocyte populations with distinct developmental states and metabolic profiles, including immature or embryonic-like cardiomyocytes that persist in specific regions during development and early postnatal stages. These populations may share functional similarities with the zebrafish primordial CMs in terms of developmental organization and maturation roles. We have now discussed this point in the Discussion and emphasized that whether a comparable lineage exists in mammals remains an important open question for future studies. We have included the discussion on page 15 of the revised manuscript.

“Although a direct corollary of *phlda2*^+^ primordial cardiomyocytes has not yet been identified in mammals, mammalian hearts contain heterogeneous cardiomyocyte populations with immature states. Whether these populations represent a functional equivalent of zebrafish primordial CMs remains an open question and needs further investigation.”

**Reviewer #3 (Public review):**

Summary:

The authors performed single-cell RNA sequencing of adult zebrafish hearts and identified markers for distinct cardiomyocyte subpopulations. One marker, phlda2, marks primordial cardiomyocytes. They generated transgenic reporter lines to characterize phlda2 expression patterns and a phlda2-NTR ablation line to determine the functional requirement of primordial cardiomyocytes during heart regeneration. They found that phlda2+ primordial cardiomyocytes are essential for myocardial morphogenesis and coronary vessel development. Interestingly, when phlda2+ primordial cardiomyocytes are ablated during heart regeneration, gata4+ cortical cardiomyocytes, coronary vessel revascularization, and scar tissue formation are not affected.

Strengths:

The authors identified a new primordial cardiomyocyte marker, phlda2. They further demonstrated that primordial cardiomyocytes are important for heart morphogenesis but dispensable for heart regeneration. Their findings reveal a potential difference between heart development and regeneration programs.

Weakness:

Despite the interesting findings, the authors did not provide supplemental data for their scRNAseq to demonstrate the data quality and support their conclusions, and some results are not well described.

We appreciate the reviewer for this important suggestion. In the revised manuscript, we have added supplemental data to support the scRNA-seq analysis, including gene expression tables for each cardiomyocyte cluster (Table S1), and full GO-term enrichment results (Table S2). In addition, we have revised the Results section to improve the clarity and description of the scRNA-seq findings. Please see detailed responses below for point-by-point clarification.

**Reviewer #3 (Recommendations for the authors):**

(1) The authors did not provide enough data to demonstrate the quality of their scRNAseq. They only mentioned that they obtained "high-quality" transcriptomics. Specific parameters such as how many total reads and reads per cell should be provided.

We thank the reviewer for this important suggestion. In the revised manuscript, we have added detailed sequencing quality metrics to the Methods section in Page 6 of the revised manuscript. The dataset contains 136,174,297 total reads with an average sequencing depth of 36,168 reads per cell.

“The newly generated scRNA-seq data yielded 136,174,297 total reads with an average sequencing depth of 36,168 reads per cell.”

(2) The authors utilized cmlc2:EGFP fish to perform scRNASeq. It will be helpful to include feature plots of cmlc2 and EGFP transcripts.

We thank the reviewer for this suggestion. We have now included the description in Page 8, and feature plots of cmlc2 transcripts and EGFP reporter expression in the scRNA-seq dataset as a supplementary figure (Fig. S1A and S1B).

“The expression of cmlc2 transcripts and EGFP reporter signal in the single-cell dataset further confirmed the enrichment of cardiomyocytes (Fig. S1A and S1B)”

(3) The authors show that notch 3 is in cluster 3 of cardiomyocytes and suggest that this reflects elevated NOTCH signaling activity. The authors might consider using RNAScope to further validate that Notch 3 is expressed in cardiomyocytes. It will be also helpful to confirm phlda2 expression patterns during zebrafish heart development and regeneration by RNAScope.

We appreciate the reviewer for this helpful suggestion. To further examine the spatial expression patterns of these genes, we analyzed publicly available spatial transcriptomic data from zebrafish hearts. We found that *phlda2* is enriched in the outer region of the heart during both uninjured and regenerating conditions. Similarly, *notch3* and *actn1* were also predominantly localized to the outer heart region in the uninjured heart. These findings are consistent with our scRNA-seq results and support the spatially restricted signature of Cluster 3 cardiomyocytes. We have included these data in Page 8, and Fig. S2A-S2C of revised manuscript.

“To further validate their spatial distribution, analysis of previously published spatial transcriptomic data revealed that *phlda2*, *notch3*, and *actn1* were predominantly expressed in the outer region of the heart (Fig.S2A-S2C).”

(4) The authors did not provide any data as supplemental tables to support their analyses of scRNAseq and GO-term analysis.

We thank the reviewer for this suggestion. In the revised manuscript, we have added new supplemental tables providing full support for the scRNA-seq and GO-term analyses (Table S1 and S2), including lists of differentially expressed genes for each cardiomyocyte cluster and the corresponding GO enrichment results.

(5) The description of the phenotype in Fig. 3A and B is not clear, especially for the sentence "trabecular muscle formation was severely impaired showing an approximately 54.7% reduction close to the ventricular wall.". The authors might consider using a bracket to show the compact muscle and trabecular muscle and the distance to the ventricular wall.

We appreciate the reviewer for this suggestion. We have added brackets in Fig. 3A to label the compact and trabecular myocardium for improved clarity. Regarding “distance to the ventricular wall,” we found that this measurement varies substantially across different regions within the same heart, making a single distance-based metric unreliable. Therefore, we quantified trabecular muscle using the trabecular area fraction (trabecular area/total ventricular area) within a defined region of interest as a robust and unbiased indicator. The reported 54.7% reduction refers to this area fraction, and we have revised the text accordingly for clarity in Page 10 of the revised manuscript.

“The compact myocardium was disorganized compared with controls, and trabecular muscle formation was severely impaired, with an approximately 54.7% reduction in trabecular area, predominantly observed in regions adjacent to the ventricular wall (Fig. 3A, 3B and S3A).”

(6) It is not clear how the authors quantify the vessel "length"/ventricular area and found that there is no difference (Fig. 3G). The vessel length is significantly shorter in the images (Fig. 3F) as the author also indicated that the vessels are fragmented.

We thank the reviewer for this important comment. Coronary vessel “length” was quantified by selecting a fixed region of interest (ROI) within the ventricular area, followed by skeletonization of *deltaC:EGFP*^+^ vessels using ImageJ. The total vessel length within the ROI was measured and normalized to the ROI area to obtain vessel length density. Although the representative images (Fig. 3F) show a more fragmented vascular pattern, the total summed vessel length within the defined region was not reduced. This indicates that primordial CM ablation primarily affects vascular organization rather than overall vessel length within the ventricular area. We have updated the figure legend for clarity of the revised manuscript

“Vessel length was measured within a fixed region of interest (ROI) after skeletonization of *deltaC:EGFP*^+^ vessels in ImageJ.”
